# Identification of structural transitions in bacterial fatty acid binding proteins that permit ligand entry and exit at membranes

**DOI:** 10.1016/j.jbc.2022.101676

**Published:** 2022-02-03

**Authors:** Jessica M. Gullett, Maxime G. Cuypers, Christy R. Grace, Shashank Pant, Chitra Subramanian, Emad Tajkhorshid, Charles O. Rock, Stephen W. White

**Affiliations:** 1 Department of Infectious Diseases, St. Jude Children’s Research Hospital, Memphis, Tennessee, USA; 2 Department of Structural Biology, St. Jude Children’s Research Hospital, Memphis, Tennessee, USA; 3 Theoretical and Computational Biophysics Group, NIH Center for Macromolecular Modeling and Bioinformatics, Beckman Institute for Advanced Science and Technology, Department of Biochemistry, and Center for Biophysics and Quantitative Biology, University of Illinois at Urbana-Champaign, Urbana, Illinois, USA

**Keywords:** fatty acid, fatty acid synthesis, fatty acid binding protein, fatty acid kinase, *Staphylococcus aureus*, phospholipids, acyl-PO_4_, acyl-phosphate, BSA, bovine serum albumin, COM, center of mass, FA, fatty acid, HMMM, highly mobile membrane mimetic MD simulation, PC, phosphatidylcholine, PG, phosphatidylglycerol, RMSD, root mean square deviation

## Abstract

Fatty acid (FA) transfer proteins extract FA from membranes and sequester them to facilitate their movement through the cytosol. Detailed structural information is available for these soluble protein–FA complexes, but the structure of the protein conformation responsible for FA exchange at the membrane is unknown. *Staphylococcus aureus* FakB1 is a prototypical bacterial FA transfer protein that binds palmitate within a narrow, buried tunnel. Here, we define the conformational change from a “closed” FakB1 state to an “open” state that associates with the membrane and provides a path for entry and egress of the FA. Using NMR spectroscopy, we identified a conformationally flexible dynamic region in FakB1, and X-ray crystallography of FakB1 mutants captured the conformation of the open state. In addition, molecular dynamics simulations show that the new amphipathic α-helix formed in the open state inserts below the phosphate plane of the bilayer to create a diffusion channel for the hydrophobic FA tail to access the hydrocarbon core and place the carboxyl group at the phosphate layer. The membrane binding and catalytic properties of site-directed mutants were consistent with the proposed membrane docked structure predicted by our molecular dynamics simulations. Finally, the structure of the bilayer-associated conformation of FakB1 has local similarities with mammalian FA binding proteins and provides a conceptual framework for how these proteins interact with the membrane to create a diffusion channel from the FA location in the bilayer to the protein interior.

Lipids are hydrophobic molecules with limited water solubility and must be transferred between membrane organelles or to soluble enzymes by specialized transfer proteins that are able to sequester their apolar cargo and facilitate their transport through the cytosol (1, 2, 3). There are five essential steps common to all lipid transfer processes (Fig. 1*A*). First, the transfer protein ferries its enclosed cargo to the membrane. Second, it collides with the membrane bilayer surface *via* an electrostatic attraction or by exploiting a specific membrane ligand such as the phospholipid head group. Third, a conformational change occurs that opens the protein interior, exposes the buried lipid, and allows for its exchange with another lipid in the membrane. A ligand-free binding protein is an obligatory intermediate in the exchange process, and some binding proteins deposit their cargo and disengage from the membrane without loading a new ligand. Fourth, the conformational change reverses and the new protein–lipid complex dissociates from the membrane. Fifth, the complex moves through the cytosol to its destination. There are detailed crystal structures of the lipid transfer proteins in solution that reveal how they sequester lipids from solvent (4, 5). In contrast, structures of the open exchange state of transfer proteins at the membrane interface remain elusive.

**Figure 1:**
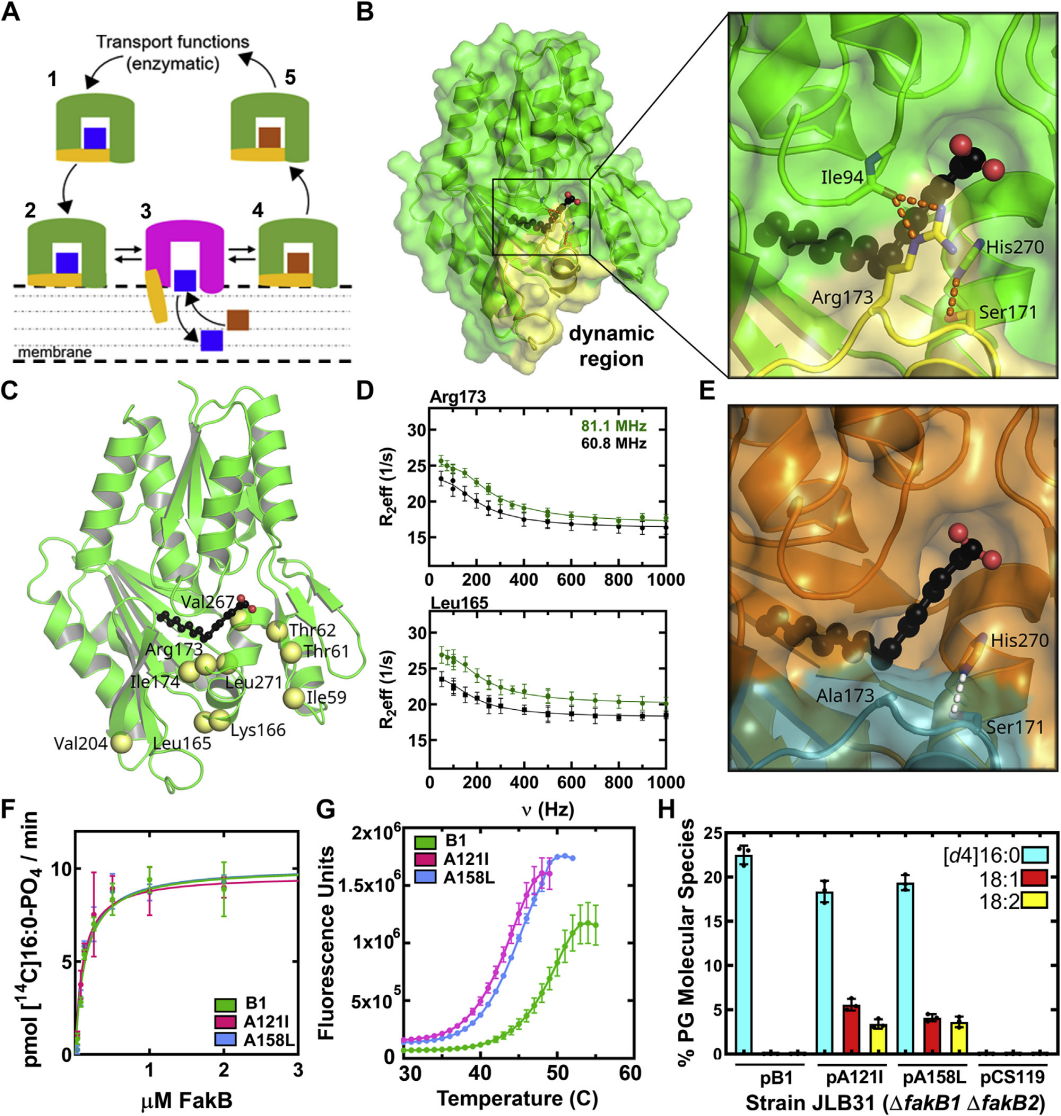
**A dynamic domain in FakB1.***A*, model for membrane lipid transfer by FakB FA binding proteins. The binding proteins exist in a closed conformation in solution (1) that sequesters a FA (*blue*) during transfer through the cytosol. The protein binds to the membrane (2), undergoes a conformational change (*magenta*) at the membrane to create a portal for FA exchange (3), and returns to the closed conformation (4) to transport a new FA (*brown*) (5). A ligand-free transfer apo-protein is an intermediate in the exchange process and in mammalian FABPs the apo-protein may deposit its cargo and disengage the membrane. *B*, FakB1 (*green*) and the dynamic region (*yellow*) form a closed conformation stabilized by hydrogen bonds (*orange dotted lines*) between Arg173 and Ser171 in the dynamic region (*yellow*) and Ile94 and His270, respectively. *C*, residues with measurable CPMG-RD exchange rates are indicated as *yellow spheres* mapped onto the FakB1 crystal structure. *D*, the ^15^N relaxation dispersion data at two field strengths for Arg173 and Leu165 were fit to a two-state model (*lines*). Error estimates for Reff were obtained from duplicate measurements at 100, 250, and 500 Hz (51). *E*, close-up of the crystal structure of FakB1(R173A) illustrating how the absence of Arg173 opens a portal into the FA. *F*, the apparent K_M_s for FakB1 and mutant FakB1 binding to FakA (FakB1, 0.13 ± 0.02 μM; FakB1(A121I), 0.1 ± 0.02 μM; FakB1(A158L), 0.13 ± 0.02 μM). Mean ± SD; *n* = 3 independent experiments. *G*, thermal denaturation curves for FakB1, 48.3 ± 0.4 °C; FakB1(A121I), 42.1 ± 0.2 °C; and FakB1(A158L), 44.0 ± 0.1 °C. *H*, substrate selectivity of FakB1, FakB1(A121I), and FakB1(A158L) *in vivo*. Mean ± SD; *n* = 3 independent experiments.

The mammalian fatty acid (FA)-binding protein (FABP) family has a common β barrel fold formed by ten antiparallel β-strands that create a large internal cavity to accommodate many different FA structures (3, 5, 6). The amino termini of FABPs form a helix-loop-helix motif that “caps” the internal cavity of the β barrel. Crystal structures show no obvious opening for an external FA to access the interior pocket, but NMR studies have revealed that the helical cap region is dynamic (5, 7, 8). Site-directed mutagenesis studies support a role for the cap helices in membrane association and the subsequent extraction of the FA (6, 7, 8, 9, 10, 11) while molecular dynamics (MD) simulations corroborate the dynamic nature of the two-helix motif in solution (12, 13, 14, 15, 16, 17, 18). The current model posits that the cap exists in an “open” state when an FABP is bound to the bilayer to allow FA exchange, but the conformation of the bilayer-associated FABP has not been described. The bacterial class of FA transfer proteins is called FakB, and the bound acyl chains are completely enclosed within the protein interior. However, they are structurally distinct from mammalian FABPs and have restricted internal cavities that are tailored to bind only selected FA structures (19, 20, 21, 22). FakB proteins shuttle FA and acyl-PO_4_ between the membrane and soluble enzymatic partners by undergoing the exchange cycle in Figure 1*A* (19, 21). *Staphylococcus aureus* has two FakBs. FakB1 binds saturated FA (19) and is responsible for the activation of FA arising from phospholipid turnover (21, 23). FakB2 selectively binds monounsaturated FA (19, 20) and is involved in the acquisition of host unsaturated FA at infection sites (24). The FA is integral to the stability of FakB proteins, and mutation of the hydrogen bond network that holds the FA carboxyl in place results in structurally unstable proteins (20). Although cellular engagement thermal shift assays indicate that apo-FakB2 exists in cells (21), the apo-FakBs are unstable and have not been isolated for biochemical analysis (19, 20, 21, 22). Like FABPs, the protein conformation that allows the FA to escape into the membrane is unknown.

Despite their dissimilar folds, the mammalian and bacterial FA transfer proteins face an identical topological challenge. Namely, a conformational change must occur to create a diffusion channel for the FA to freely exchange between its position in the membrane bilayer and the interior of the transfer protein. Here, we use a combination of X-ray crystallography, NMR spectroscopy, MD simulations, site-directed mutagenesis, and functional biochemical assays to characterize the membrane-bound conformation of FakB. This study focuses on FakB1, an FA transfer protein from *S. aureus* that binds palmitic acid (16:0) before presenting it to FakA for phosphorylation and exchange with the membrane. NMR dynamics measurements detect a dynamic 23-residue region covering the FakB1 FA binding tunnel. We captured crystal structures of the open FakB1 conformation by introducing point mutations that insert bulky residues into the narrow FA tunnel. The open conformation arises from a conformational change in the dynamic region that rotates helix α8 outward to uncover the FA-binding tunnel and reorganizes the adjacent α8-β9 loop into a new amphipathic helix. MD simulations reveal how the newly formed helix α8′ inserts into the phospholipid bilayer to create a diffusion path for the FA to exit into the membrane. Site-directed mutagenesis coupled with biochemical assays corroborates the roles of key residues in this process. These data provide an understanding of how a conformational change in FakB1 facilitates the exchange of FA between the protein interior and the membrane that provides key insights into how this process occurs in mammalian FA binding proteins.

## Results

### A dynamic region in FakB1

FakB crystal structures show that the FA is completely buried within the protein interior with only the FA carboxylate group exposed for phosphorylation (20, 21, 22). The structures also suggest a potential focal point for the conformational exchange that must occur to release the enclosed FA. The bulk of FakB1 forms the acyl chain binding pocket with the region between Asp164 and Lys186 forming a lid over the first eight carbons of the FA chain (Fig. 1*B*). Residues 175 to 186 are generally poorly resolved in the many deposited FakB structures, and in FakB1, this region has the highest B-factors (21) suggesting that these residues are conformationally flexible. This region is connected to the rest of the protein by a hydrophobic interface and terminates at the bound FA with two hydrogen-bonding interactions between the side chains of Arg173 and Ser171 and the backbone carbonyl of Ile94 and the Nδ1 of His270, respectively (Fig. 1*B*). We used NMR spectroscopy to independently identify the dynamic regions of FakB1 in solution. The 2D [^15^N,^1^H] TROSY spectrum of [^15^N,^13^C]FakB1 at 293 K showed well-dispersed resonances, and all but four residues were assigned with the aid of spectra collected at 293 K using [^2^H,^15^N,^13^C]FakB1 (Fig. S1). The secondary structure calculated from TALOS analysis of the NMR spectra correlates well with the secondary structural elements observed in the FakB1 crystal structure (Fig. S2*A*) with the exception of helix α1 and sheet β5 that are not apparent from the TALOS analysis. Also, residues 178 to 181 within the α8-β9 loop are in an α-helical conformation based on the NMR spectra but have a less pronounced helical nature in the crystal structure.

Carr–Purcell–Meiboom–Gill relaxation dispersion (CPMG-RD) spectra were acquired using [^2^H,^15^N,^13^C]FakB1, at two different field strengths (81.1 MHz and 60.8 MHz) at 293 K to map the mobility within the FakB1 structure. The ^15^N relaxation dispersion profiles revealed that FakB1 is stably folded but ten residues adjacent to the bound FA are clearly dynamic (Fig. 1*C*). Four of these residues are within the Asp164-Lys186 region and include the central Arg173 and Leu165 at the amino-terminus of the region. The datasets were a close fit to a two-state exchange model using the equation of Carver and Richards (25) (Fig. 1*D*). Data from all ten residues were used to fit a global two-state model with forward (k_f_) and reverse (k_r_) exchange rate constants 12.0 ± 1.0 s^−1^ and 753.0 ± 53.0 s^−1^, respectively, yielding an exchange rate constant (k_ex_ = k_f_ + k_r_) of 764.2 ± 53.0 s^−1^. A Gibbs free energy barrier of 2.41 ± 0.07 kcal/mol is calculated from the exchange rates for the FakB1 conformational change. The major (98.5%) and the minor (1.5%) conformations exchange on the millisecond timescale.

The rotation of the dynamic Arg173 residue away from the protein is predicted to expose and promote the opening of a portal to the buried FA. We determined the X-ray structure of FakB1(R173A) to determine if this was the case (Fig. 1*E*), but its overall structure was identical to that of FakB1 (RMSD = 0.210 Å) (Fig. S3*A*). The hydrophobic interface and the Ser171-His270 interaction are apparently sufficient to maintain the connection between the dynamic region and the body of FakB1 even though the absence of Arg173 does expose the first eight carbons of the FA to solvent as predicted (Fig. 1*E*). FakB1(R173A) is a few degrees less stable than FakB1 consistent with the loss of the hydrogen bond connection to Ile94 (Fig. S3*B*), and as expected (20), FakB1(R173A) was catalytically inactive.

### X-ray crystallography captures an open conformation

Unlike the mammalian FABPs, FakB1 has a tight binding tunnel that presents an alternative opportunity to induce the conformational change by using site-directed mutagenesis to modulate the optimal packing of the FA into its binding site. We identified two alanine residues (121 and 158) in the tunnel and mutated them to bulkier residues to partially occlude the FA-binding pocket and push the FA out of its pocket to destabilize the fully closed FakB1 conformation (Fig. S3*C*). The FakB1(A121I) and FakB1(A158L) mutant proteins were fully active in FA kinase assays with the same apparent affinity for FakA (Fig. 1*F*), and analytical ultracentrifugation verified that both continue to form tight complexes with FakA (Table S1). However, the mutations did alter two key properties of FakB1. First, compared with the wild-type protein, the thermal stabilities of FakB1(A121I) and FakB1(A158L) were reduced by 6 °C and 4 °C, respectively (Fig. 1*G*), showing that the mutations indeed prevent FakB1 from adopting its most stable conformation. Second, the mutations altered the FA selectivity of FakB1 in a physiological setting. When expressed in *Escherichia coli*, the total amount of FA incorporated by the FakB1 mutant proteins was the same as the wild-type protein, but FakB1 only supported the incorporation of palmitic acid (16:0), whereas both mutant proteins were less specific and also incorporated oleate (18:1) and linoleate (18:2) (Fig. 1*H*). These data show that the tunnel mutations alter the stability and selectivity of FakB1, but do not impair the overall function of FakB1 *in vivo* or *in vitro*.

**Figure 2:**
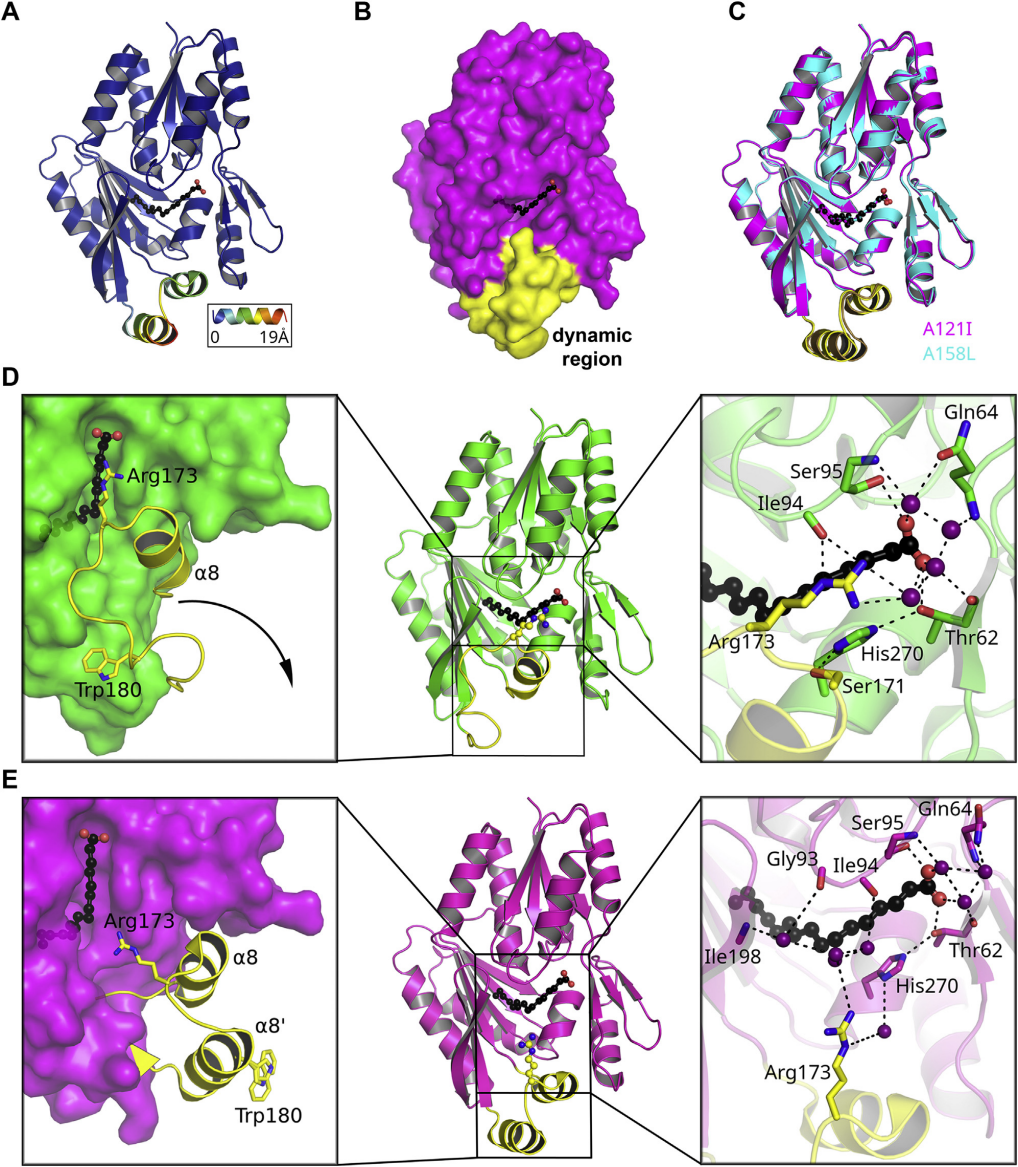
**The “closed” and “open” states of FakB1.***A*, the RMS deviations (represented by *blue* (low) to *red* (high)) in the main chain Cα positions of the FakB1(A121I) crystal structure relative to wild-type FakB1. *B*, surface rendering of FakB1(A121I) showing how the rotation of the dynamic region (*yellow*) away from FakB1 (*magenta*) creates a portal. *C*, overlay of the FakB1(A121I) and FakB1(A158L) crystal structures. *D*, domain interactions in FakB1. *Left Panel*, the dynamic region (*yellow*) is packed tightly against FakB1 (*green*). Hydrophobic residues in the α8-β9 loop (dynamic region) make van der Waals interactions with FakB1 and the Arg173 at the end of helix α8 is closed. *Right Panel*, the water-mediated hydrogen bond network (*black dotted lines*) between Arg173 and Ser171 on the dynamic region (*yellow*) and Ile94 and His270 (*green*), respectively. The Ser171-His270-Thr62-FA carboxylate-Ser95 hydrogen bond network and structured waters (*purple spheres*) at the FA carboxyl binding site are shown. *E*, domain interactions in FakB1(A121I). *Left Panel*, movement at the base of helix α8 rotates it away and the α8-β9 loop is released from FakB1 (*magenta*) to form helix α8′. *Right Panel*, the conformational change disrupts the Ser171-His270-Thr62-FA carboxylate-Ser95 hydrogen bond network and Ile94, Arg173, and His270 form contacts with surrounding water molecules (*purple spheres*).

The crystal structure of FakB1(A121I) was determined at 2.02 Å resolution (Table 1). We observed two molecules (molA and molB) in different conformational states in the asymmetric unit. The prototypical FakB1 closed conformation was exemplified by molB, but in molA, the dynamic region undergoes a significant structural rearrangement (Fig. 2*A*). Specifically, Arg173 disengages from the FA and Ile94, helix α8 (Leu165 to Ser171) moves away from the protein, and residues 175 to 186 form a new α-helix that rotates away from the protein by ∼180° in concert with the movement of helix α8. Excluding the structural rearrangements observed in FakB1(A121I), the open and closed structures superimpose with an RMSD of 0.356 Å showing that the conformational change is confined to these residues (Fig. 2*A*). Notably, the conformational change occurs precisely in the region of the protein revealed by NMR spectroscopy to exhibit dynamic properties (Fig. 1*C*). The surface rendering of FakB1(A121I) shows that the conformational change exposes the ligand-binding cavity and creates a portal to the FA (Fig. 2*B*).

**Table 1:** X-ray crystallography data collection, refinement, and validation statistics

Complex	FakB1(A121I)-palmitate (open)	FakB1(A158L)-myristate (open)	FakB1(R173A)-palmitate (closed)	FakB1(A121I, A158L)-Palmitate (open)
PDB codes	6MH9	6NM1	7SCL	7SG3
Data collection				
Beamline (APS)	SER-CAT 22-ID	SER-CAT 22-ID	SER-CAT 22-ID	SER-CAT 22-ID
Temperature (K)	100	100	100	100
Space group	P1	P1	P1	P1
Cell dimensions				
a, b, c (Å)	33.5, 53.9, 86.0	33.4, 53.5, 86.2	33.2, 53.9, 84.4	33.2, 53.6, 85.9
α, β, γ (°)	103.7, 90.4, 107.3	76.9, 89.4, 72.3	104.3, 90.7, 107.5	104.2, 90.5, 107.0
Resolution (Å)	83.29–2.02	83.74–2.33	49.60–1.60	49.46–2.35
*R*_sym_ or *R*_merge_	0.066 (0.726)^a^	0.086 (0.813)	0.056 (0.596)	0.069 (0.492)
*R*_*pim*_	0.039 (0.438)	0.051 (0.475)	0.033 (0.351)	0.042 (0.298)
Unique reflections	34,458 (2509)	23,124 (2250)	63,745 (2951)	20,941 (1997)
Redundancy	3.8 (3.7)	3.9 (3.9)	3.8 (3.8)	3.6 (3.6)
CC (1/2)	0.997 (0.724)	0.998 (0.748)	0.998 (0.841)	0.997 (0.867)
Mean I/σI	12.3 (1.9)	10.2 (1.9)	7.0 (2.0)	6.9 (1.9)
Completeness (%)	94.4 (91.3)	98.2 (97.7)	89.7 (83.5)	92.0 (90.7)
Wilson B-factor (Å^−2^)	34.8	38.0	24.0	42.3
Model quality				
*R*_work_/*R*_free_	20.7/25.8	23.7/28.8	16.19/19.31	21.17/25.09
No. atoms				
Protein	4539	4483	4426	4188
Ligand/ion	36	32	48	36
Water	207	134	359	69
*B*-factors				
Protein	41.7	53.6	39.9	58.2
Fatty acid	36.0	48.7	35.1	61.3
Water	46.3	51.4	48.0	53.6
R.m.s. deviations				
Bond lengths (Å)	0.002	0.002	0.008	0.003
Bond angles (°)	0.449	0.477	1.022	0.500
Ramachandran plot				
Favored (%)	97.0	92.1	96.8	97.2
Allowed (%)	2.7	6.0	2.5	2.0
Outliers (%)	0.4	1.9	0.7	1.3
Clashscore	5.7	7.8	5.4	6.7

a Values in parentheses are for highest-resolution shell.

We also determined the 2.33 Å crystal structure of FakB1(A158L) (Table 1), which was structurally identical to FakB1(A121I). The open FakB1(A158L) molA structure superimposes on the FakB1(A121I) molA structure with an RMSD of 0.265 Å (Fig. 2*C*). In FakB1(A121I), the bulkier side chain displaces the middle of the aliphatic chain of the FA by 0.9 Å and its distal end by ∼1.4 Å that, in turn, shifts the tunnel residues Val162, Leu191, and Leu271 and pushes against the dynamic region. A similar process occurs in FakB1(A158L). The 2.35 Å crystal structure of the FakB1(A121I, A158L) double mutant was also determined (Table 1), and superposition of the FakB1(A121I) and FakB1(A121I, A158L) structures shows that they are basically identical with an RMSD = 0.241 Å (Fig. S3*D*). Thus, three independent mutants generated the identical conformational change. The FakB1(A121I) is the highest-resolution crystal structure and was used as the representative open conformation for further analysis.

The dynamic region between residues 177 and 186 is generally poorly resolved or not present in many FakB structures deposited in the PDB. In our FakB1 structure (21), residues 180 to 183 have the highest B-factors in the crystal structure, but the positions of the residues in the α8-β9 loop can be located in the 2Fo-Fc and SA-OMIT electron density maps (Fig. S4*A*). In the FakB1(A121I) structure, the α8-β9 loop residues become helix α8′, and the residues within this helix have lower B-factors than in the FakB1 structure (Fig. S4*B*). This allows the conformation of the new 12-residue helix α8′ to be clearly defined in the 2Fo-Fc and SA-OMIT maps (Fig. S4*B*).

In the closed conformation, Trp180 is tightly packed against the hydrophobic interior of the protein, and Arg173 is packed onto the bound FA (Fig. 2*D*, left panel). There is also a hydrogen bond network consisting of Thr62, Ser95, Ser171, His270, and the FA carboxylate that appears to balance the negative charge on the FA (Fig. 2*D*, right panel). In the open conformation, Arg173 becomes disengaged from FakB1 (Fig. 2*E*, left panel), and the newly formed 12-residue helix that we designate as α8′ has a distinct exposed hydrophobic surface consisting of Ala177, Trp180, Val181, Leu184, and Leu185. We suggest that the low dielectric constant within the FakB1(A121I) crystal lattice coupled with the location of Phe38 and Ile44 on the neighboring molecule that is only available to molA create an apolar environment for the relocation and stabilization of helix α8′ (Fig. S5*A*). We also note that the disengagement of Arg173 from the FA and the outward movements of α8 and α8′ disrupt the hydrogen bond network surrounding the FA carboxylate by breaking the key Ser171-His270 hydrogen bond and exposing His270 to solvent (Fig. 2*E*, right panel).

### FakB1(A121I) dynamics

FakB1(A121I) was analyzed by NMR to determine how the mutation impacts protein dynamics. The 2D [^15^N,^1^H] TROSY spectrum of FakB1(A121I) was similar to that of the wild-type protein (Fig. S5*B*) with the largest chemical shift differences localized to the area adjacent to the Ala121 mutation (Fig. S5*C*). CPMG-RD data for [^2^H,^15^N,^13^C]FakB1(A121I) collected at two field strengths at 293 K revealed 12 residues with ^15^N relaxation dispersion profiles that mapped to the same locations as the ten dynamic residues in FakB1 (Fig. S5*D*). These residues are more mobile than in FakB1(121I) as exemplified by Arg173 and Leu165 (compare Figs. S5*E* and 1*D*). The global exchange rate was k_ex_ = 757.3 ± 42.0 s^−1^, with forward (k_f_) and reverse (k_r_) exchange rate constants, 36.0 ± 3.0 s^−1^ and 722.0 ± 42.0 s^−1^, respectively. The population of the minor species (p_minor_) increased from 1.5% in FakB1 to 5% in FakB1(A121I). The lower Gibbs free energy barrier (ΔG = 1.75 ± 0.02 kcal/mol) is consistent with the more abundant FakB1(A121I) minor state in solution.

**Figure 3:**
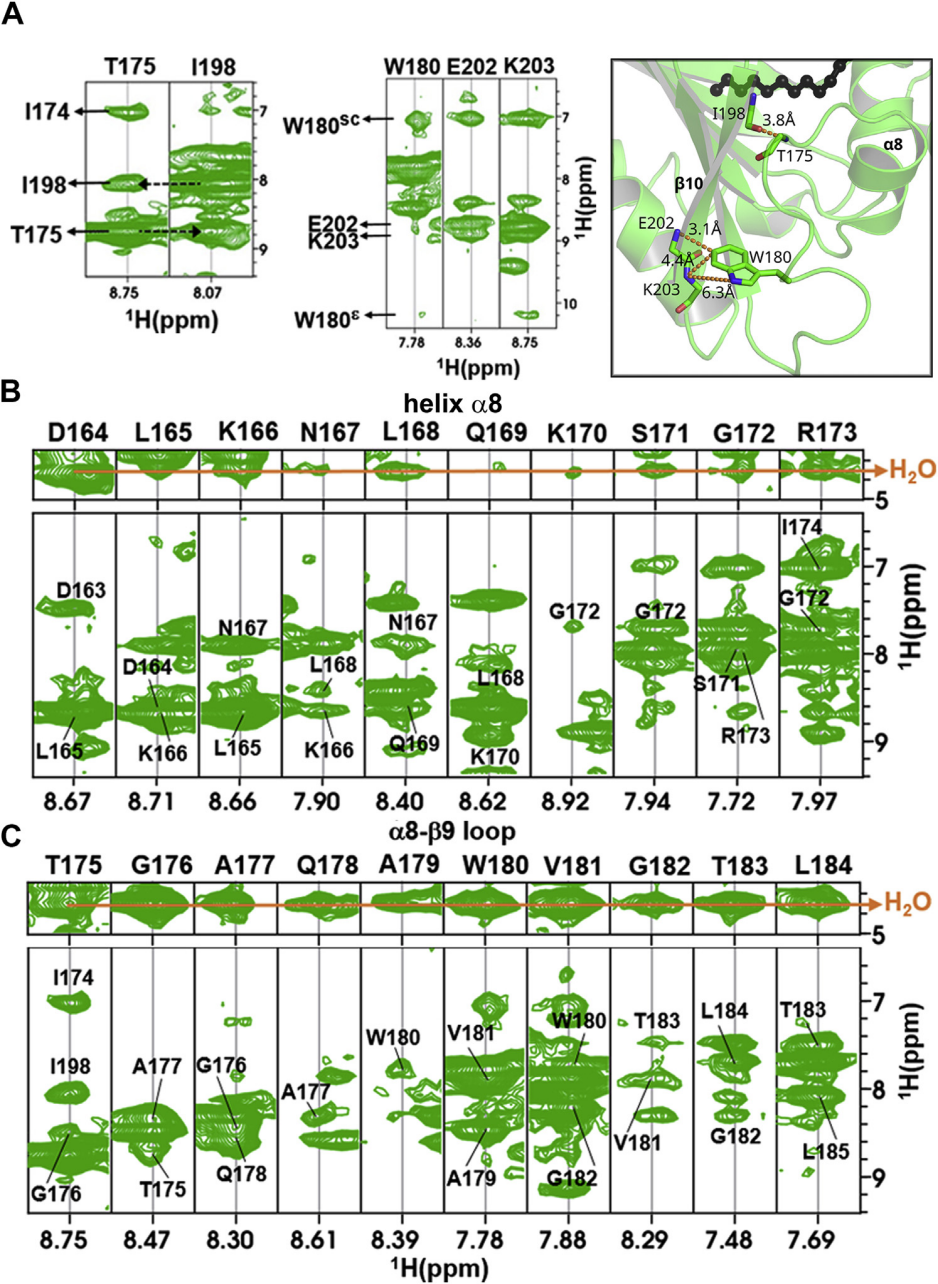
**Solution conformation of FakB1.***A*, NOE contacts between residues in the α8-β9 loop of the dynamic region and residues in FakB1 are consistent with the closed FakB1 crystal structure. The NOE interactions (*dotted orange lines*) and computed distances from the NMR data are shown in the *right panel* (sc, side chain protons; ε, amide protons). *B*, sequential NOEs confirm the existence of helix α8 in solution. The *orange line* indicates exchange cross peaks with water. *C*, the water cross-peaks from Thr175-Leu184 (*orange line*) are indicative of a structured α8-β9 loop, but the sequential NOEs centered on Trp180-Val181 suggest partial helical character in this region.

Although the NMR experiments show that FakB1 exchanges between major and minor conformations in solution, the data do not establish the structures of the major and minor species. We therefore analyzed specific ^N^H-^N^H NOEs in the ^15^N-resolved NOESY spectrum. The high quality of the NOEs was confirmed by the strong ^N^H^i^-^N^H^i+1^ and ^N^H^i^-^N^H^i+2^ NOEs for helix α4 in both FakB1 (Fig. S6*A*) and FakB1(A121I) (Fig. S6*B*). The presence of clear signature NOEs in both FakB1 (Fig. 3*A*) and FakB1(A121I) (Fig. S7*A*) within the dynamic region that undergoes the conformational change confirms that the major species corresponds to the closed conformation. First, the Thr175-^N^H to Ile198-^N^H NOE and the reverse Ile198-^N^H to Thr175-^N^H NOE show that Thr175 on the α8-β9 loop is in close proximity to Ile198 on strand β10. Second, NOEs between the side chain aromatic protons of Trp180 to Glu202-^N^H and Lys203-^N^H confirm that Trp180 adopts its buried location observed in the closed conformation and not the exposed location observed in the open conformation. Taken together, the NOE data are consistent with the closed conformation as the major state in solution for FakB1 and its mutant derivatives. We attempted to identify weaker NOEs that would support that the minor population corresponds to the open crystal conformation but were unable to do so.

Sequential NOEs from Asp164 to Arg173 in FakB1 are consistent with the existence of helix α8 in solution. However, exchange cross-peaks with water in Asp164-Lys166 and Ser171-Arg173 suggest that helix α8 is slightly unraveled at both ends (Fig. 3*B*). In addition, although the Thr175-Leu184 α8-β9 loop residues have water cross-peaks consistent with the loop conformation seen in the X-ray structure (Fig. 3*C*), the sequential NOEs across this region also suggest that the loop has some helical characteristics centered on Trp180-Val181. The crystal structure of FakB1 reveals that there is indeed a turn between residues Gly176 and Val181 with helical characteristics (Fig. 2*D*, left panel). These observations are confirmed by the analysis of the NMR spectra of helix α8 (Fig. S7*B*) and the α8-β9 loop (Fig. S7*C*) of FakB1(A121I). Taken together, the NOE data are consistent with the closed conformation as the major state in solution for FakB1 and its mutant derivatives.

### FakB1 membrane interactions

**Figure 4:**
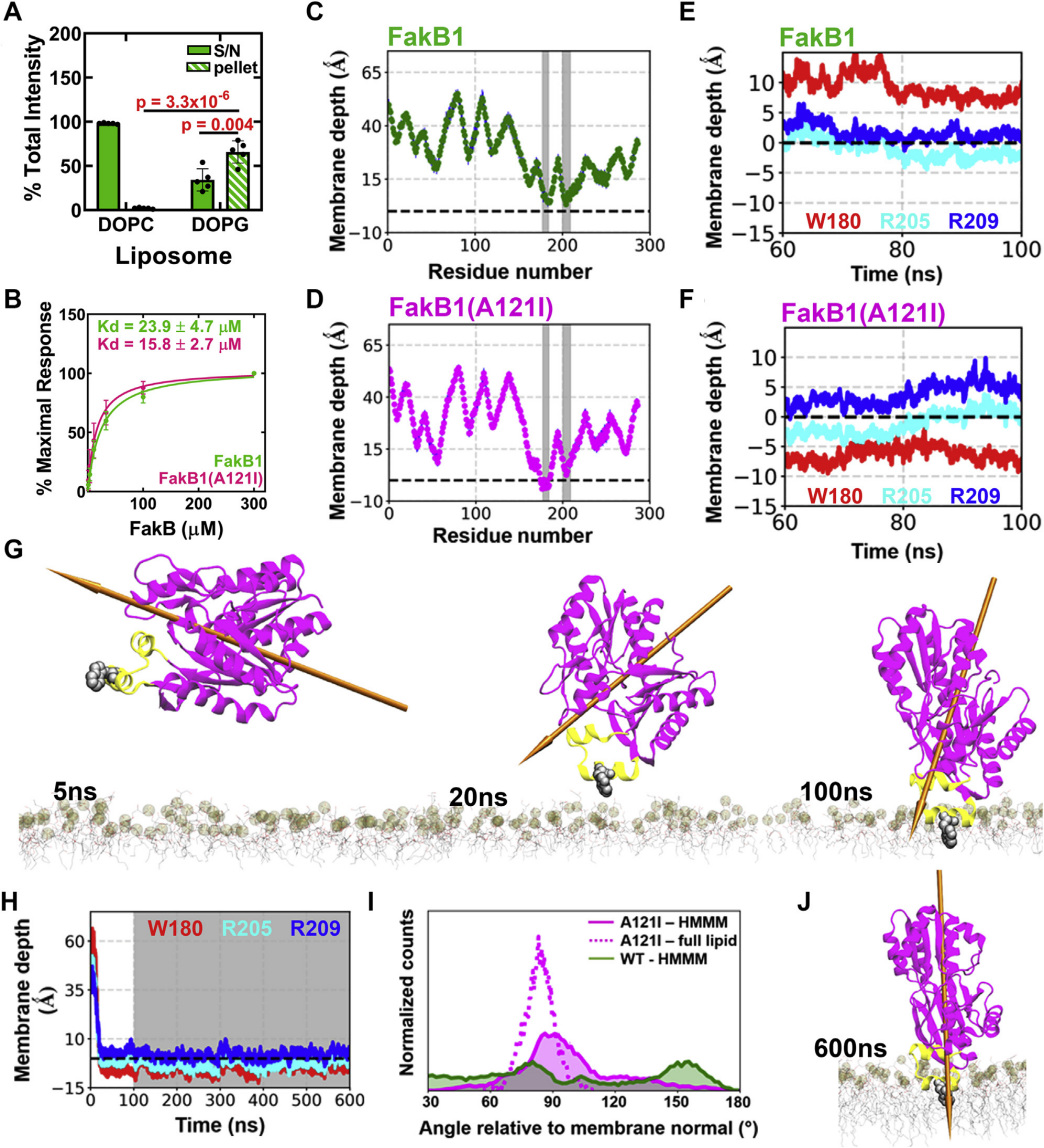
**The open conformation of FakB1 inserts into the PG bilayer.***A*, FakB1 association with PG, but not PC, liposomes was assessed using a liposome pull-down experiment. Mean ± SD is shown, *n* = 5 independent experiments. Two-tailed Student’s *t* tests were used to evaluate statistical significance. *B*, SPR analysis of FakB1 and FakB1(A121I) showing similar binding kinetics using PG:PC vesicles (50/50). Kd values were calculated from four independent experiments for each protein. Best fit values ±SE are shown. *C* and *D*, representative ensemble-averaged location of Cα residues along the membrane normal (z) axis of the PG bilayer calculated during last 50 ns of the ten HMMM membrane-binding simulations. The phosphate layer of the lipid bilayer is set as a reference at z = 0 shown with a *dashed line*. *Gray bars* represent residues adjacent to or penetrating the bilayer. *C*, FakB1. *D*, FakB1(A121I). *E* and *F*, locations of the center of masses (COM) of Trp180, Arg205, and Arg209 side chains along the membrane normal (z axis) of the PG bilayer (*dotted black line*) plotted during the last 40 ns of HMMM membrane binding simulations. *E*, FakB1. *F*, FakB1(A121I). *G*, snapshots of the HMMM simulation of FakB1(A121I) at the indicated times show the evolution of the binding event and the changing orientation of the FakB1(A121I) dipole moment towards the vertical (*arrow*). *H*, time evolution of (COM) distances for Trp180 (*red*), Arg205 (*cyan*), and Arg209 (*blue*) with respect to the membrane normal (*dotted black line*) (z = 0). A HMMM membrane binding simulation was performed for the first 100 ns (*white background*) capturing FakB1(A121I) binding to the membrane within the first 20 ns, followed by a “full-lipid” simulation for additional 500 ns (*gray background*) showing the stability of the bound configuration. *I*, FakB1(A121I) binds with helix α8′ positioned at a ∼90° angle relative to the membrane normal (z axis) that is most pronounced in the ‘full-lipid’ simulation. *J*, the FakB1(A121I) protein dipole moment is aligned with the z axis of the membrane bilayer in the “full-lipid” simulation.

FakB1 must bind to the membrane and create a path for the FA to travel from its location in the phospholipid bilayer (26) to the protein interior, and vice versa. Our structural and dynamic analyses strongly suggest that an open conformation of FakB1 mediates this process, and we tested this hypothesis by investigating the binding of FakB1 to charged phospholipids. Phosphatidylglycerol (PG) is the major phospholipid in the inner bilayer of the *S. aureus* membrane, and PG liposome pull-down experiments reveal that FakB1 has an affinity for PG, but not for phosphatidylcholine (PC) liposomes (Fig. 4*A*). We then determined the affinities of FakB1 and FakB1(A121I) for PC:PG (50/50) vesicles using PC vesicles as a negative control in surface plasmon resonance (SPR) assays. We detected a 15 to 20 μM affinity of both FakB1 and FakB1(A121I) for PG-containing vesicles (Fig. 4*B*), which is similar to its micromolar affinity for FakA and consistent with its transport function. Binding to PC layers was not detected.

We developed a linear, coupled biochemical assay using FakA to measure the FakB1 FA exchange/transport activity. The phosphorylation of [^14^C]16:0 in a PG liposome as substrate yielded a transfer rate of 1.4 ± 0.2 nmol/min/μg, whereas the transfer rate was two orders of magnitude slower (10.7 ± 0.6 pmol/min/μg) when the [^14^C]16:0 was presented as a bovine serum albumin (BSA) complex (Fig. S8*A*). These data confirm that FakB1 is designed to efficiently extract FA from PG bilayers rather than to exchange with free or protein-solubilized FA.

### Molecular dynamics (MD) simulations of FakB1 binding to membrane and FA release

MD simulations employing the HMMM (highly mobile membrane mimetic) model of the membrane (27, 28, 29, 30, 31, 32) were used to study the interaction of FA-bound FakB1 with the membrane. The FA was modeled as the protonated species. HMMM uses short-tailed lipids to significantly enhance the mobility of lipids, thereby facilitating spontaneous protein insertion into a bilayer. Ten replicates of each MD simulation condition were obtained by initially placing the protein 10 Å away from the bilayer surface in a random orientation. Simulations of the closed conformation with PG show that FakB1 consistently orients its electropositive surface, centered on Arg205, toward the electronegative PG bilayer and then engages the membrane (Figs. 4*C* and S8*B*). Although this membrane association is consistently observed in multiple simulation replicates, it is not intimate, as evidenced by the ensemble-averaged Cα positions of Trp180, Arg205, and Arg209, being above the phosphate plane by 6.6 ± 1.9 Å, 5.5 ± 0.9 Å, and 9.0 ± 0.8 Å, respectively (Figs. 4*C* and S8*B*). In contrast, simulations with the FakB1(A121I) open conformation consistently show residues 177 to 184 (α8′) penetrating the bilayer. Although the Cα carbons of Arg205 and Arg209 are in similar locations relative to the phosphate plane in the open and closed conformation simulations (compare Fig. 4*C* with Fig. 4*D*), the Cα carbon of Trp180 has flipped from 6.6 Å above the phosphate plane in the closed conformation to 3.1 ± 1.2 Å below the phosphate plane in the open conformation of the protein (Figs. 4*D* and S8*C*). Simulations using PC-only bilayers did not show any membrane association for the open conformation (Fig. S8*D*).

Given the large size of the arginine and tryptophan side chains, the positions of Cα atoms do not fully capture the penetration of these residues into the membrane. To determine the depth of membrane insertion, we monitored the time evolution for the center of masses (COM) of the side chain atoms of Trp180, Arg205, and Arg209 in membrane-binding simulations (Fig. S9, *A*–*C*). In the closed state, the Trp180 and Arg209 side chains are 9.0 ± 2.4 Å and 2.9 ± 1 Å, respectively, above the phosphate plane of the bilayer, whereas the Arg205 side chain reaches the phosphate layer (−0.13 ± 0.9 Å) (Fig. 4*E*). In the open state, the Arg209 side chain remained 3.4 ± 1.6 Å above the phosphate plane, and Arg205 continues to engage the phosphate layer (1.6 ± 1.6 Å). However, the Trp180 side chain is deeply buried in the bilayer at a depth 6.5 ± 1.2 Å below the phosphate layer (Fig. 4*F*). These data show that Arg205 and the surrounding electropositive surface play a key role in initially orienting the protein and engaging the bilayer in both the closed and open conformations. However, the final location of Trp180 in the membrane-bound complexes is very different with its center of mass shifting ∼12 Å in the transition between the closed and open conformations.

Snapshots of FakB1(A121I) during a typical HMMM membrane-binding simulation show that it orients properly with respect to the membrane and engages it within 20 ns; subsequently, the protein forms a stable membrane-associated complex with the PG bilayer between 20 and 100 ns (Figs. 4*G* and S9*A*). The HMMM approach was validated by performing a “full-lipid” MD simulation of the FakB1(A121I) open conformation that extended to 500 ns beyond the HMMM membrane-binding simulations. This full-lipid simulation maintained a stable association of the open conformation with the PG bilayer with the same mode and depth of membrane insertion, as measured by monitoring the locations for the center of masses for Trp180, Arg205, and Arg209 (Fig. 4*H*). There is a more pronounced orientational preference for membrane docking of helix α8′ in the full-lipid simulation, with helix α8′ positioned parallel to the phosphate plane and the protein dipole moment aligned with the membrane normal (z axis) (Fig. 4, *I* and *J*). Thus, both the HMMM and full tail simulations capture stable membrane-bound states for the open conformation and provide the same insights into the interaction mode of the protein with the PG bilayers.

### Validation of the MD model

**Figure 5:**
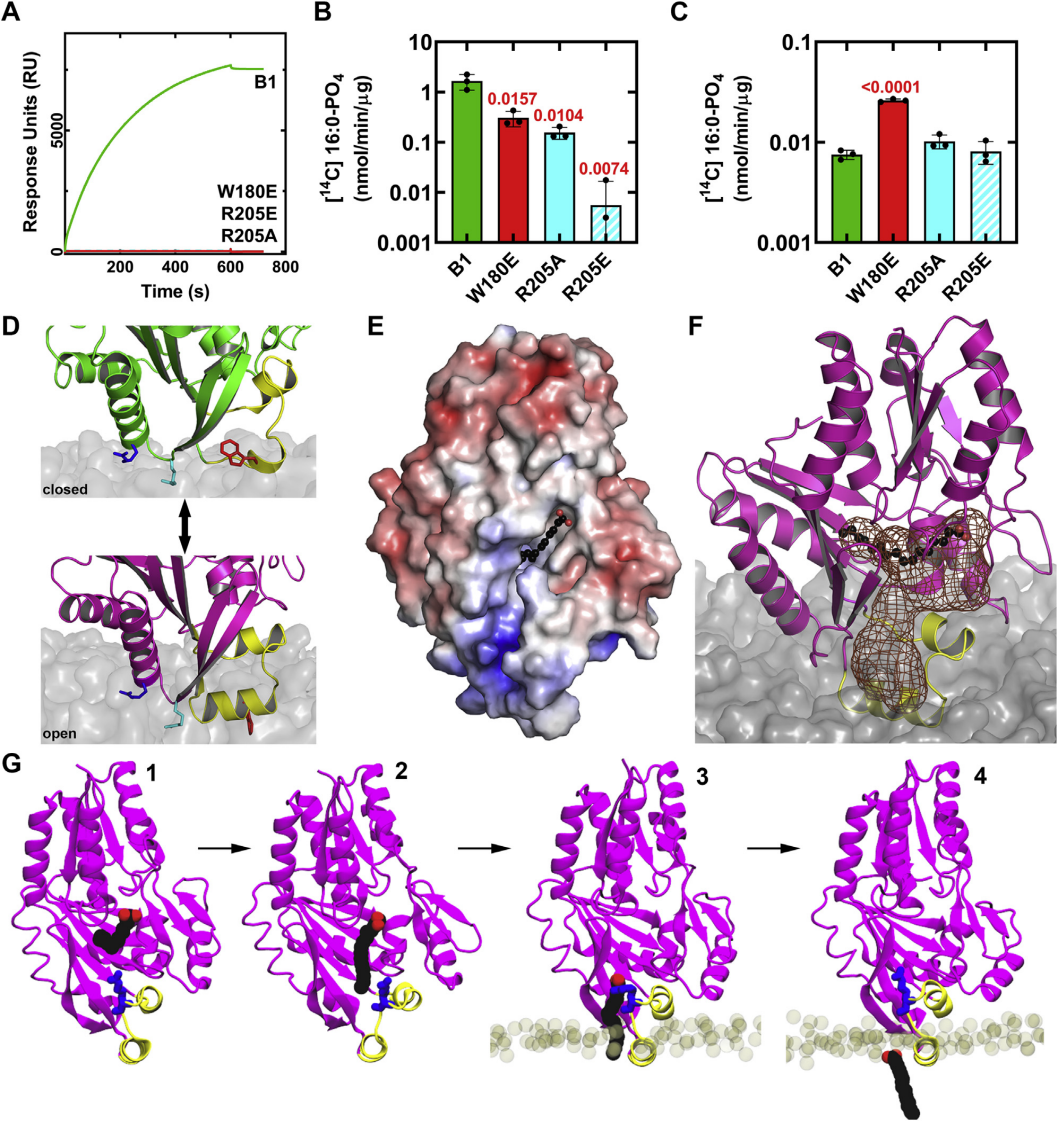
**Role of Arg205 and helix α8′ in FakB1 membrane interactions.***A*, representative SPR sensograms of FakB1, FakB1(R205E), FakB1(W180E), and FakB1(R205A) binding to PC:PG (50/50) vesicles. *B*, FakB1 exchange assay using [^14^C]16:0 presented in PG liposomes. *C*, FakB1 FA exchange assay using [^14^C]16:0 presented as a BSA-16:0 complex. Statistical significance was determined using a two-tailed Student’s *t* test and comparing mutant protein values to wild-type FakB1. Mean values ±SD are shown; *n* = 3 independent experiments. *D*, close-up of the reversible conformational changes allowing FakB1 association and disengagement from a PG bilayer. *Top*, Trp180 (*red*) of the closed conformation is packed against the protein, the Arg205 (*cyan*) side chain interacts with the phosphate layer and the Arg209 (*blue*) side chain lies along the bilayer surface. *Bottom*, the open conformational change inserts helix α8′ with Trp180 pointing into the center of the bilayer and helix α8′ oriented parallel to and below the phosphate plane of the bilayer. Arg205 interacts with the phosphate groups of the PG bilayer, and Arg209 associates with the bilayer surface. *E*, electrostatic charge (*red*, negative to *blue*, positive) surface rendering of the open FakB1(A121I) crystal structure shows that a surface groove is created from the FA binding site to the electropositive end of the protein. *F*, mesh (chocolate) shows the groove becomes a diffusion channel that extends from the FA binding site in the protein interior to the phosphate plane of the bilayer when it is embedded in the membrane. *G*, snapshots from the FakB1(A121I) Movie S1 showing the four key steps in FA release into the bilayer: (1) The beginning of the simulation with the FA in its binding site. (2) The FA tail moves out of its deep pocket and into the space created by the outward rotation of helix α8. (3) The FA carboxyl is released from the hydrogen bond network and the FA slides down the tunnel interacting with Arg173 (*blue*). (4) The FA diffuses into the bilayer.

The MD simulations strongly suggest that Trp180 and Arg205 are key residues in membrane binding of the protein. We therefore prepared FakB1(R205A), FakB1(R205E), and FakB1(W180E) mutants to experimentally test this conclusion. FakB1 was not substantially destabilized by these mutations (Fig. S9*D*), but SPR experiments showed that all three mutant proteins were defective in binding to PG vesicles (Fig. 5*A*). Furthermore, the mutants showed reduced FA exchange activity compared with wild-type FakB1 when [^14^C]16:0 was presented in a PG liposome (Fig. 5*B*). In contrast, when [^14^C]16:0 was delivered in BSA, the wild-type and mutant proteins were all active although the FakB1(W180E) exchange rate is higher in this assay (Fig. 5*C*). Thus, all mutant proteins were able to mediate catalysis using BSA-solubilized FA but were defective in acquiring FA from a liposome. These data strongly support the roles of these residues in membrane binding as suggested from the MD simulations.

The MD simulations posit a model where the electropositive surface of FakB1 surrounding Arg205 initially orients the closed conformation with respect to the membrane surface with Arg205 poised to interact with the phosphate layer (Fig. 5*D*). The displacement of Arg173 and the formation of helix α8′ trigger membrane penetration of the open conformation and the stabilization of helix α8′ within the bilayer (Fig. 5*D*). Analysis of the open FakB1(A121I) crystal structure reveals a groove created by the conformational change that forms a channel extending from the FA-binding site to the electropositive end of the protein (Fig. 5*E*). In the simulations, this channel becomes partially buried when the open conformation is bound to a PG bilayer to create a hydrophobic diffusion path for the FA to travel from the protein interior to the phosphate plane of the bilayer (Fig. 5*F*). The details of this process are revealed in one of the FakB1(A121I) MD simulations in which the FA disengages from the binding pocket, travels down the diffusion path created by insertion into the bilayer to insert the FA tail into the hydrocarbon layer and release the FA carboxyl at the phosphate plane of the bilayer (Movie S1). This process consists of four steps (Fig. 5*G*). The FA tail that is initially bound in the deep FakB1 hydrophobic pocket first moves into the portal created by the rotation of helix α8 in the open conformation. The FA carboxyl then disengages from the weakened hydrogen bond network and slides down the tunnel along a hydrophobic surface of FakB1. The FA journey is interrupted for a short time by its interaction with Arg173, but is then released to insert into the membrane where it can diffuse within the plane of the bilayer. A separate, membrane-associated FA can then enter FakB1 by the reverse process.

## Discussion

### Dynamic region of FakB switches conformations

FakB has a dynamic region adjacent to the FA-binding pocket that was suggested by FakB crystal structures and verified by NMR analyses. These dynamic properties render the region metastable and poised to switch conformations when it encounters a lipid bilayer that stabilizes the newly formed hydrophobic helix α8′. In solution, FakB oscillates between two states, a major state (>95%) that corresponds to the closed X-ray structure and a minor state (<5%) that we could not structurally characterize that encompasses the residues involved in the transition to the open structure. The FakB1(A121I) and FakB1(A158L) crystal structures capture the open conformation that is generated by the introduction of bulkier side chains into the FA-binding pocket and stabilized by the crystal packing architecture that creates a surrogate hydrophobic environment that favors the formation of helix α8′. Trp180 is a sentinel hydrophobic residue in FakB1 whose significant conformational change and deep insertion into the membrane, captured by MD simulations, illustrate how the open conformation is anchored into the membrane.

### Roles of Arg173 and Arg205

Arg173 and Arg205 are completely conserved in the FakB protein family and are essential for multiple FakB functions. Arg205 is at the center of the positively charge surface patch that forms the initial membrane encounter site in the MD simulations, and its side chain maintains contact with the bilayer phosphate groups in both the closed and open conformations. Arg205 is also the key residue responsible for the binding of FakB to FakA (20). Arg173 is required in the FA kinase catalytic mechanism (20) precluding the biochemical evaluation of its role in FA exchange by site-directed mutagenesis. However, MD simulations suggest that Arg173 orients the FA with respect to the entrance to the diffusion channel and may escort the FA to and from the bilayer. Arg173 is also appropriately positioned to act as a “lure” dangling just above helix α8′ to capture an FA located at the phosphate zone of the bilayer and facilitate its movement into the protein’s binding pocket before the reloaded FakB assumes the closed conformation and separates from the membrane. We propose that Arg173 acts as a molecular latch that must be opened to allow the exit of the FA from the closed conformation. Arg173 covers the bound FA and provides a key hydrogen bond connection with the body of the protein. Although the removal of Arg173 is not sufficient to generate the open conformation in the crystal lattice, its disengagement is essential for the conformation change to occur.

### Similarities to mammalian FABPs

**Figure 6:**
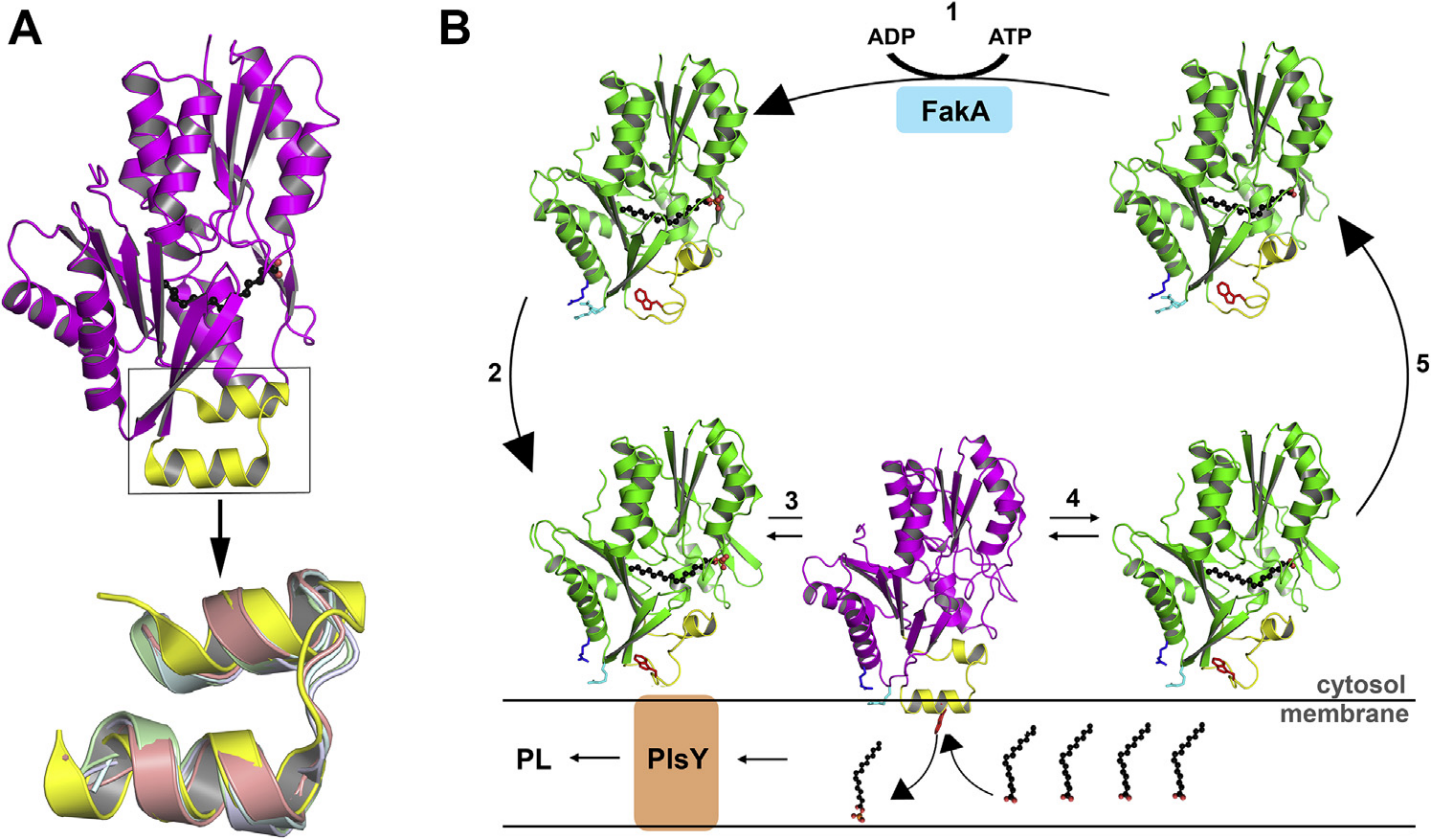
**FA exchange at the bilayer interface.***A*, the two-helix motif in the FakB1 open conformation is overlaid with the two-helix motifs present in mammalian FA-binding proteins. FakB1(A121I) (PDB: 6MH9), *yellow*; human adipocyte FABP4 (PDB: 1TOW), *salmon*; murine adipocyte FABP4 (PDB: 1A2D), *cyan*; and rat intestinal FABP2 with palmitate (PDB: 2IFB), *green*. *B*, conformational cycling model. (1) Phosphorylation of the FA carboxylate bound to the closed FakB1 conformation in the cytosol. (2) The electropositive surface of FakB1 is attracted to the electronegative PG membrane surface; R205 (*cyan*), R209 (*blue*), and W180 (*red*) are shown. (3) Dynamic region (*yellow*) transitions to the open conformation and helix α8′ inserts below and parallel to the phosphate plane of the bilayer with Trp180 (*red*) fully inserted. This FakB1-PG interaction creates a diffusion channel for FA allowing the bound acyl-PO_4_ to exchange with another FA in the bilayer. (4) Dynamic region transitions back to the closed conformation and FakB1 separates from the membrane (5) to carry its cargo to FakA to initiate another cycle of FA transfer/exchange. The *red arrow* shows the location of the membrane-docked two-helix motif.

The two-helix membrane insertion motif in the FakB1 open conformation has intriguing parallels with the two-helix domain in mammalian FABPs (5, 33). X-ray structures show that the prototypical FABP has two domains: a large β-barrel bottle that holds the FA attached by a hinge to a helix-turn-helix domain that caps the bottle. Although none of the solution or crystal structures have captured an open FABP conformation, it is hypothesized that the FA enters and exits the bottle *via* the rotation of the two-helix cap motif (8, 34, 35, 36). MD simulations with FABP4 and a membrane surface (37, 38) show that the two-helix bundle associates with the membrane *via* electrostatic interactions, which is supported by extensive mutagenesis and cross-linking experiments (7, 8, 39). NMR dynamics measurements suggest that an opening between the β-sheets may function as an alternate portal (18, 40), but it is pointing away from the bilayer in the MD simulations. The membrane penetrating two-helix bundle in the open FakB1 conformation overlays perfectly with the two-helix bundle in FABPs (Fig. 6*A*) suggesting that the FABP4 helices may function in a similar manner to the FakB1 two-helix bundle by rotating away from the bottle opening and penetrating into the membrane to create a diffusion path for the FA. Thus, the structurally distinct FakB1 and FABP4 may both utilize a two-helix bundle to interact with the bilayer to solve the identical topological problem faced by FA-binding proteins.

## Conclusions

This study reveals the molecular details of the membrane-bound FakB conformation that leads to a model for the FakB FA exchange cycle (Fig. 6*B*). A key feature of the closed FakB conformation is the positively charged patch surrounding Arg205 that orients the protein with respect to the negatively charged PG bilayer and associates with the bilayer surface through the electrostatic interaction of the Arg205 side chain with the phosphate groups of PG. The surface-associated FakB converts to the open conformation that tightly docks to the membrane by the insertion of the new helix α8′ below the phosphate plane of the bilayer. This conformational change creates a diffusion path for the bound acyl-PO_4_/FA to release into the bilayer and for FakB to accept another FA. FA exchange is a rapid process that is the basis for the accumulation of acyl-PO_4_ in the FA kinase assay (19, 20, 21, 22) and is most efficient when the FA is presented to the enzyme system within a PG liposome (Fig. 5, *B* and *C*). Ligand-free FakB is an intermediate in this cycle. There is evidence that apo-FakB exists in cells (21), but it has proved too unstable to isolate for biochemical analysis (20). Thus, the transition to the open conformation in the bacterial FA-binding proteins achieves four essential mechanistic goals: it opens a portal to the FA-binding pocket, disrupts the hydrogen-bonding network that holds the FA carboxylate in place to facilitate its release, forms a new α-helix that inserts into the bilayer, and creates a diffusion channel between the protein interior and the phosphate plane of the bilayer where FA resides. The reloaded FakB protein then returns to the closed conformation, dissociates from the membrane, and delivers its cargo to FakA for another round of phosphorylation.

## Experimental procedures

### Bacterial strains and reagents

Sources of supplies were: PerkinElmer, [^14^C]16:0 (specific activity, 56.1 mCi/mol); Millipore-Sigma, all FA; Cambridge Isotope Laboratories, Inc, 7,7,8,8-tetradeuteriohexadcanoic acid ([*d*_4_]16:0) and algal amino acid mixture (U-^13^C, U-D, U-^15^N); Sigma Aldrich, all reagents for buffers; Fisher Scientific, Casamino acids; New England Biolabs, restriction enzymes; Agilent, QuikChange Lightning Site-Directed Mutagenesis Kit; anti-polyHistidine−alkaline phosphatase antibody (Sigma). High-capacity streptavidin agarose beads (Thermo Fisher). The bacterial strains and their origins are listed in Table S2. The FakA expression plasmid (pJLB11) and the FakB1 expression plasmid (pCS106) were previously constructed (19). Expression vectors containing FakB1(A121I), FakB1(A158L), FakB1(W180E), FakB1(R205E), FakB1(R205A) FakB1(R173A), and FakB1(A121I A158L) were generated using QuikChange Lightning Site-Directed Mutagenesis Kit. pCS106 was used as a template for all constructs except FakB1(R173A) and FakB1(A121I A158L), which used pPJ631 or pPJ632, respectively (Table S2). FakB1(A121I) For and FakB1(A121I) Rev primers were used to generate the A121I mutant, pPJ583. FakB1(A158L) For and FakB1(A158L) Rev primers were used to generate the A158L mutant, pPJ584 (Table S2). B1 W180E primers 1 and 2 were used to generate the W180E mutant, pPJ593. B1 R205A primers 1 and 2 generated the R205A mutant, pPJ594. B1 R205E primers 1 and 2 generated the R205E mutant, pPJ595 (Table S2). SaFakB1(R173A) F and SaFakB1(R173A) R were used to generate the R173A mutant, pPJ631, and B1(A121I A158L) F and B1(A121I A158L) R were used to generate the A121I A158L double mutant, pPJ632 (Table S2). Manufacturer’s protocol was followed for mutagenesis PCR. PCR products from the mutagenesis underwent Dpn-I digestion and transformation into XL10-Gold ultracompetent cells (carbenicillin 50 μg/ml). Purified plasmids were verified by sequencing. The QuikChange Lightning Site-Directed Mutagenesis Kit was also used to create A121I, A158L, and W180E FakB1 mutants in the pCS119 vector used in metabolic labeling experiments. The *fakB1* gene in pCS119 (pB1) was previously created (22) and used as a template. CO A121I F and CO A121I R primers were used to generate the A121I mutant in pCS119 (pA121I). CO A158L F and CO A158L R primers were used to generate the A158L mutant in pCS119 (pA158L). CO B1 W180E Fwd and CO B1 W180E Rev primers were used to generate the W180E mutant in pCS119 (pPJ597) (Table S2).

### Protein expression and purification

Expression plasmids containing *S. aureus* FakA, wild-type FakB1, or FakB1 mutants (A121I, A158L, W180E, R205E, R205A, R173A, A121I A158L) were transformed into BL21 DE3 *E. coli* electrocompetent cells (Invitrogen) and purified as previously described (19). Briefly, induced cells were lysed and centrifuged, and the supernatant was incubated at room temperature with 10 mM fatty acid (palmitate for all constructs except FakB1(A158L) where myristate was used) for 1 h to exchange a known fatty acid into the binding pocket before purification (19, 20, 21, 22). This step is required to load apo-FakB1 with a known FA and to remove the mixture of FA picked up in the expression system. This allows for a homogeneous FakB1 preparation with only a single FA. For heavy labeled protein, colonies from the previously mentioned transformation were grown at 37 °C in minimal media containing carbenicillin (50 mg/ml), glucose (2 g/l), and Casamino acids (2 g/l). Overnight cultures were reinoculated (A_600_ = 0.01) into 500 ml fresh media containing glucose (2 g/l) and either double or triple labeled algal amino acids (U-^13^C, U-D, U-^15^N) (2 g/l) instead of Casamino acids to express either [^15^N,^13^C]FakB1 or [^2^H,^15^N,^13^C]FakB1. Cultures grew until an A_600_ = 0.7 before being induced with IPTG (1 mM) and shaken overnight at 16 °C. Cells were harvested and purified as previously described (19).

### Protein crystallization and structure determination

Crystals were grown in 0.1 M MES/imidazole pH 6.5, 0.03 M sodium nitrate, 0.03 M disodium hydrogen phosphate, 0.03 M ammonium sulfate, 12.5% PEG 1000, 12.5% PEG 3350, and 12.5% MPD. These conditions are similar to those of FakB1, and the crystal parameters are also very similar (21). Crystals were cryoprotected with the mother liquor containing 30% glycerol and flash frozen in liquid nitrogen prior to data collection. Diffraction data (360°) were integrated with XDS (41, 42) and scaled using AIMLESS/CCP4 (43). The structures were solved by molecular replacement using MOLREP/CCP4 (44) and the wild-type structure as the search model (PDB: 5UTO). In the molecular replacement solutions, residues 163 to 186 in one of the two molecules in the crystal asymmetric unit were incorrect and were rebuilt into omit maps using ITERATIVE BUILD OMIT MAP/PHENIX (44) The final structures were obtained by cycles of rebuilding and refinement using COOT (45) and PHENIX.REFINE (46). Water molecules were placed both automatically and manually with COOT. In all refinements, 5% of the reflections were excluded for calculation of the R_free_ values. The final models were deposited to the PDB database and the molecule and refinement parameters are shown in Table 1. We used PyMOL/ALIGN to produce the structural alignments and RMSD calculations. In Figure 6*A*, the alignment was performed using only the two helical segments from each of the proteins. The alignment in Figure 2*A* was performed using the CCP4/SUPERPOSE program.

### NMR sample preparation and data collection

All samples were prepared in an NMR buffer comprising 20 mM Tris, 200 mM NaCl (pH 7.5), 90% H_2_O, 10% D_2_O with 1 mM *S. aureus* wild-type FakB1 or FakB1(A121I). NMR data were collected on Bruker Avance 600 MHz, 700 MHz, and 800 MHz spectrometers equipped with TCI triple-resonance cryogenic probes initially analyzed at 313 K using [^15^N,^13^C]FakB1 to narrow the lines and make the TROSY-based 3D structural assignments. [^2^H,^15^N,^13^C]FakB1 was used at 293 K to study dynamics. These included HNCA, HNCACB, CBCA(CO)NH, HNCO, and HN(CA)CO along with 2D TROSY ^1^H-^15^N HSQC. A TROSY-based ^15^N-resolved [^1^H,^1^H]-NOESY spectrum was also recorded (mixing time of 120 msec). These data allowed 90% of the backbone resonances to be assigned. These assignments were transferred to a 293 K spectrum, and the assignments were confirmed using a 3D HNCA spectrum. [^2^H,^15^N,^13^C]FakB1 was used to collect TROSY-based 3D HNCA, HNCACB, HN(CO)CA spectra along with a TROSY-based ^15^N-resolved [^1^H,^1^H]-NOESY spectrum (mixing time of 150 msec at 293 K). Assignment of [^2^H,^15^N,^13^C]FakB1(A121I), was confirmed using a 3D HNCA along with a TROSY-based ^15^N- resolved [^1^H,^1^H]-NOESY spectrum recorded (mixing time of 150 msec at 293 K at 850 MHz). All the data were processed using BRUKER Topspin version 4.0.5, NMRPipe (v7.9) (47) and analyzed using CARA (v1.8.4). All spectra were referenced directly using DSS for the ^1^H dimension, and ^13^C and ^15^N frequencies were referenced indirectly. The secondary structure predictions were made using backbone and side chain torsion angles with the TALOS-N program (48).

### NMR relaxation dispersion data collection and analysis

^15^N single quantum CPMG (Carr–Purcell–Meiboom–Gill) relaxation dispersion experiments (49, 50) were used to study the exchange processes in FakB1 and FakB1(A121I). Relaxation dispersion datasets were recorded on both samples at 293 K using both 600 and 800 MHz spectrometers. Fourteen CPMG frequencies from 50 Hz to 1000 Hz were sampled during a constant-time relaxation interval of 40 ms with an interscan delay of 3 s, including a reference spectrum without the constant-time relaxation interval. Amide resonance intensities were converted into effective relaxation rates (49, 50) and plotted as a function of the CPMG frequency. Error estimates for R_eff_ were obtained from duplicate measurements at 100, 250, and 500 Hz frequencies as previously described (51). The relaxation dispersion data were fit to extract global exchange parameters, including exchange rates and population of the states, along with residue specific values such as ^15^N chemical shift differences between exchanging states and intrinsic ^15^N relaxation rates, R_2,0_. This was accomplished with ShereKhan (52) using Carver–Richards equation (25) for two-site slow exchange. We also used the equations of McConnell (53) to calculate the exchange rates, which were not significantly different from the values obtained by the Carver-Richards method. Residues that did not overlap with other resonances (Ile59, Thr61, Thr62, Leu165, Lys166, Arg173, Ile174, Val204, Val267, Leu271) were used in the exchange parameter calculation for FakB1. For FakB1(A121I), residues Ile59, Thr61, Thr62, Ser63, Leu165, Arg173, Val204, Gly264, Val267, Leu271, Gly272, and Leu276 were used in the calculation.

### Thermal stability

The structural stabilities of FakB1 and mutant derivatives A121I, A158L, W180E, R205E, R205A, R173A, and A121I A158L were evaluated using thermal shift assays. Five microliters of protein (200 μm) was added to a total of 95 μl of 200 mM MgCl_2_, 150 mM NaCl, 20 mM HEPES (pH 7.5), 2 mM ATP, and Sypro Orange (1:200) (Invitrogen). The plate was centrifuged at 1500 rpm for 2 min before being placed in an Applied Biosciences 7500 RealTime PCR instrument. A thermal scan from 25 °C to 95 °C was performed using an increment rate of 1 °C/min. The first derivative values of each temperature were calculated and graphed as a function of temperature. All experiments were performed in triplicate.

### Analytical ultracentrifugation

Sedimentation velocity experiments were conducted in a ProteomeLab XL-I analytical ultracentrifuge (Beckman Coulter) following standard protocols unless mentioned otherwise. Samples in buffer containing 20 mM Tris-HCl, pH 8, 200 mM NaCl, 10 mM EDTA were loaded into cell assemblies comprised of double sector charcoal-filled centerpieces with a 12 mm path length and sapphire windows. Buffer density and viscosity were determined in a DMA 5000 M density meter and an AMVn automated micro-viscometer (both Anton Paar), respectively. The cell assemblies, containing identical sample and reference buffer volumes of 390 μl were placed in a rotor and temperature equilibrated at rest at 20 °C for 2 h before it was accelerated from 0 to 50,000 rpm. Rayleigh interference optical data as well as absorbance data at 280 nm were collected at 1 min intervals for 12 h. The velocity data were modeled with diffusion-deconvoluted sedimentation coefficient distributions c(s) (54). The s-value was corrected for time, and finite acceleration of the rotor was accounted for in the evaluation of Lamm equation solutions (55). Isotherm data of the signal-average s-values, sw(c), of the total sedimenting system derived from integration of the complete c(s) distributions of all FakA (A) and FakB1 (B) mixtures of sedimentation velocity data at concentrations stated in Table S1 were fitted to a mixed self- and heteroassociation model; (A + A) + B + B forming complexes (AA), AB, (AA)B, (AA)BB; self-association of A with two symmetric sites for two B’s. The association scheme used in this analysis was (A + A) + B + B ↔ A +AB + B↔ (AA)B +B↔(AA)(B)2 with the dimer dissociation constant KDAA for the self-association of A and KDAB for the heterointeraction of A and B with two symmetric sites. Errors of the fits represent the 68.3% confidence interval (CI) using an automated surface projection method (56). Calculations were performed using SEDFIT/SEDPHAT (https://sedfitsedphat.nibib.nih.gov/software/default.aspx). All plots were created in GUSSI (57) (http://biophysics.swmed.edu/MBR/software.html).

### FA kinase assays

FA kinase assays contained 0.1 M Tris-HCl (pH 7.5), 10 mM ATP, 20 mM MgCl_2_, 0.2 μm FakA, 20 μM of [^14^C]16:0, Triton X-100 (1%), and fatty acid-free BSA (1 mg/ml). The indicated concentrations of purified FakB proteins were in a total volume of 60 μl. Tubes were incubated at 37 °C for 20 min before acetic acid (0.6%) was added, and 40 μl was pipetted on a DE81 Whatman filter paper disc and discs were washed three times, for 20 min each, in ethanol containing acetic acid (1%). Discs were dried and counted by scintillation counting. Apparent Km values were determined performing a nonlinear regression and using the Michaelis–Menten equation. Acyl-phosphate production was also assessed by spotting reactions mixtures on thin layer plates. Reactions contained 0.1 M Tris-HCl, pH 7.5, 10 mM ATP (pH 7.5), 20 mM MgCl_2_, either 200 μM liposomes (900 μM DOPG, 10% [^14^C]16:0) or fatty-acid free BSA (1 mg/ml) with [^14^C]16:0 (20 μM), and FakB1 (1 μM for BSA, 0.005 μM for liposomes). The mix was incubated at room temperature for 15 min before FakA (4 μM) was added to begin the reaction. Reagents were incubated at 37 °C for 20 min before acetic acid (0.8%) was added and reactions ceased. Ten microliters of each reaction was spotted on a Silica Gel H plates (Analtech) that were developed with ethanol:chloroform:triethylamine:water (34:30:35:6.5) and then imaged on a Typhoon FLA 9500. The bands were quantified using ImageQuant software. Statistical significance was determined using the two-tailed Student’s *t* test.

### FA incorporation experiments

*S. aureus* strain JLB31 with a plasmid containing a wild-type *fakB1* gene, a mutant (pA121I, pA158L), or empty plasmid (pCS119) was inoculated into 5 ml of Luria broth containing 0.1% Brij-58, and tubes were shaken at 37 °C until an A_600_ of 0.5 was reached. An FA mixture (10 μM each) of [*d*_4_]16:0, 18:1, and 18:2 was added to the culture and incubated at 37 °C for 30 min before lipids were extracted. All experiments were performed in triplicate and representative spectra are shown. Lipid extracts were resuspended in chloroform:methanol (1:1). PG was analyzed using a Shimadzu Prominence UFLC attached to a QTrap 4500 equipped with a Turbo V ion source (Sciex). Samples were injected onto an Acquity UPLC BEH HILIC, 1.7 μm, 2.1 × 150 mm column (Waters) at 45 °C with a flow rate of 0.2 ml/min. Solvent A was acetonitrile, and solvent B is 15 mM ammonium formate, pH 3. The HPLC program was the following: starting solvent mixture of 96% A/4% B, 0 to 2 min isocratic with 4% B; 2 to 20 min linear gradient to 80% B; 20 to 23 min isocratic with 80% B; 23 to 25 min linear gradient to 4% B; 25 to 30 min isocratic with 4% B. The QTrap 4500 was operated in the Q1 negative mode. The ion source parameters for Q1 were: ion spray voltage, −4500 V; curtain gas, 25 psi; temperature, 350 °C; ion source gas 1, 40 psi; ion source gas 2, 60 psi; and declustering potential, −40 V. The system was controlled by the Analyst software (Sciex). The sum of the areas under each peak in the mass spectra was calculated, and the percent of each molecular species present was calculated with LipidView software (Sciex). Incorporation of [*d*_4_]16:0 was calculated by combining the values of [*d*_4_]16:0, [*d*_4_]18:0, and [*d*_4_]20:0 molecular species, the values of 18:1 and 20:1 were combined for 18:1 incorporation; the values of 18:2 and 20:2 were combined for 18:2 incorporation. All experiments were performed in triplicate.

### Biotinylated liposome pull-down assay

Five microliters of purified FakB1 (40 μM) was added to 20 μl of high-capacity streptavidin agarose beads (Thermo Fisher) and 25 μl of liposomes containing the following: 1 mol percent of DOPE-N-(cap biotinyl) and 99 mol percent of DOPC or DOPG; 1 mol percent of DOPE-N-(cap biotinyl), 89 mol percent DOPG, and 10 mol percent of [^14^C]16:0; 1 mol percent of DOPE-N-(cap biotinyl), 79 mol percent DOPG, and 20 mol percent of [^14^C]16:0. Buffer (0.1 M Tris-HCl, pH 7.5) was added to a total volume of 200 μl, and samples were incubated at room temperature on a shaker for 1 h before being centrifuged at 2000*g* for 5 min. Supernatant was decanted without disturbing the streptavidin beads, and excess moisture was wicked from the beads before resuspending in an equal volume of buffer. A Western blot was performed on aliquots from the supernatant and pellet using monoclonal anti-polyHistidine−alkaline phosphatase antibody (Sigma). Blots were images on a Typhoon FLA 9500, and band intensities were quantified using ImageQuant (Cytiva). Statistical significance was determined using the two-tailed Student’s *t* test.

### Surface plasmon resonance (SPR)

SPR experiments were conducted at 25 °C on a Biacore T200 optical biosensor (Cytiva Life Sciences) using the methods of Del Vecchio and Stahelin (58). A 50:50 mixture of DOPG:DOPC was used for the variable component vesicles, and 100% DOPC was used for the control vesicles allowing for the measurement of net binding to DOPG. Dried lipids (0.5 mM) were hydrated overnight in binding buffer (20 mM Tris-HCl, pH 7.5, 200 mM NaCl) prior to extrusion. Extruded lipids were captured on an equilibrated, preconditioned L1 chip (Cytiva), and the chip was blocked with 3 to 4 injections of fatty-acid free BSA (0.1 mg/ml). Proteins were prepared in binding buffer as a threefold dilution series with maximum concentration of 100 μM and injected for 600 s at a flow rate 10 μl/min. The lipid surfaces were regenerated between cycles with NaOH (50 mM) injected for 12 s at flow rate 50 μl/min. The data were processed, double-referenced, and analyzed using the software package Scrubber2 (version 2.0c, BioLogic Software). Equilibrium dissociation constants (*K*_D_) were determined by fitting the data to a 1:1 (Langmuir) equilibrium affinity model.

### Molecular dynamic simulations

Crystal structures of FakB1•16:0 (PDB: 5UTO) and FakB1(A121I)•16:0 (PDB: 6MH9) were used as initial structures for the simulations. PSFGEN plugin of VMD (Visual Molecular Dynamics) (59) was used to add a C-terminal carboxylate capping group, an N-terminal ammonium capping group, and hydrogen atoms. The proteins were placed, individually, in a water box using the SOLVATE plugin of VMD. The solvated system was neutralized with Na^+^ and Cl^−^ ions (0.15 M NaCl) using the AUTOIONIZE plugin of VMD. The FA was modeled as protonated. The molecular system was then energy minimized for 2000 steps and equilibrated for 1 ns. The final equilibrated protein was used for all the consequent membrane-binding simulations. HMMM membranes were employed to capture the membrane-binding conformations of FakB1•16:0 and FakB1(A121I)•16:0. The short-tailed lipids used in the model enhance lipid diffusion and membrane reorganization, thereby allowing spontaneous peripheral protein insertion within the timescales of the simulations (27, 60, 61). All the independent HMMM membranes were constructed using HMMM BUILDER in CHARMM-GUI (62, 63). Multiple membrane-binding simulations of FakB1•16:0 and FakB1(A121I)•16:0 were performed in the presence of pure PG or PC lipid bilayers and each simulation ran for 100 ns. All membrane-binding simulations began with the protein in the aqueous solution at least 10 Å away from the *cis*-leaflet phosphate plane. The orientation of each protein was varied with respect to the membrane normal (z) axis to ensure the final membrane-bound conformation was not biased by the initial placement.

To test the stability of the membrane-bound conformation of the protein obtained from HMMM simulations (62, 63) a membrane-bound replica of FakB1(A121I)•16:0 was converted to full membrane (full-lipid) using CHARMM-GUI. After another round of equilibration following the CHARMM-GUI protocol, the entire membrane-bound FakB1(A121I)•16:0 was simulated for 500 ns. All simulations were performed in NAMD2 (64, 65) using CHARMM36m protein and lipid force fields and TIP3P water (66, 67). Nonbonded interactions were calculated with a 12 Å cutoff and a switching distance of 10 Å. Long-range electrostatic interactions were calculated using the particle mesh Ewald (PME) method (68). The temperature was maintained at 310 K by Langevin dynamics with a damping coefficient of 1.0 ps^−1^. Short-tailed HMMM lipids are best simulated in a fixed area ensemble (27, 31). The pressure was therefore maintained only along the membrane normal (NPnAT) using the Nosé–Hoover Langevin piston method (69).

All analyses were performed in VMD (59). The membrane-binding configuration and depth of protein insertion into the lipid bilayer were measured by averaging the z positions of all Cα-atoms with respect to the *cis*-leaflet phosphate plane, over the last 50 ns of HMMM simulations. The protein was considered membrane-bound if helix α8′ (residues 177–184) penetrated the bilayer. A heavy-atom cutoff of 3.5 Å was used to define specific lipid–protein contacts. We also monitored the time evolution of the center of mass (COM) of the side chain heavy atoms of W180, R205, and R209.

## Data availability

The structural coordinates have been deposited as PDB codes 6MH9, 6NM1, 7SCL, and 7SG3. The NMR data are deposited into the Biological Magnetic Resonance Data Bank under the entry IDs 50555 (FakB1) and 50556 (FakB1(A121I)). All study data are included in the article or supporting information.

## Supporting information

This article contains supporting information (19, 21, 23, 70).

## Conflict of interest

The authors declare they have no conflicts of interest with the contents of this article.
