# Supplementary material for: Identification of structural transitions in bacterial fatty acid binding proteins that permit ligand entry and exit at membranes
Source: J Biol Chem. 2022 Feb 3;298(3):101676. doi: 10.1016/j.jbc.2022.101676 (PMC8892158; doi:10.1016/j.jbc.2022.101676)
Supplement: Supplemental Figures S1–S9, Tables S1 and S2 [file mmc2.pdf]

Supplementary Materials

Identification of Structural transitions in bacterial fatty acid binding proteins that permit ligand entry and exit at membranes

Jessica M. Gullett, Maxime G. Cuypers, Christy R. Grace, Shashank Pant, Chitra Subramanian, Emad Tajkhorshid, Charles O. Rock and Stephen W. White

CONTENTS

Figures S1-S9

Tables S1-S2

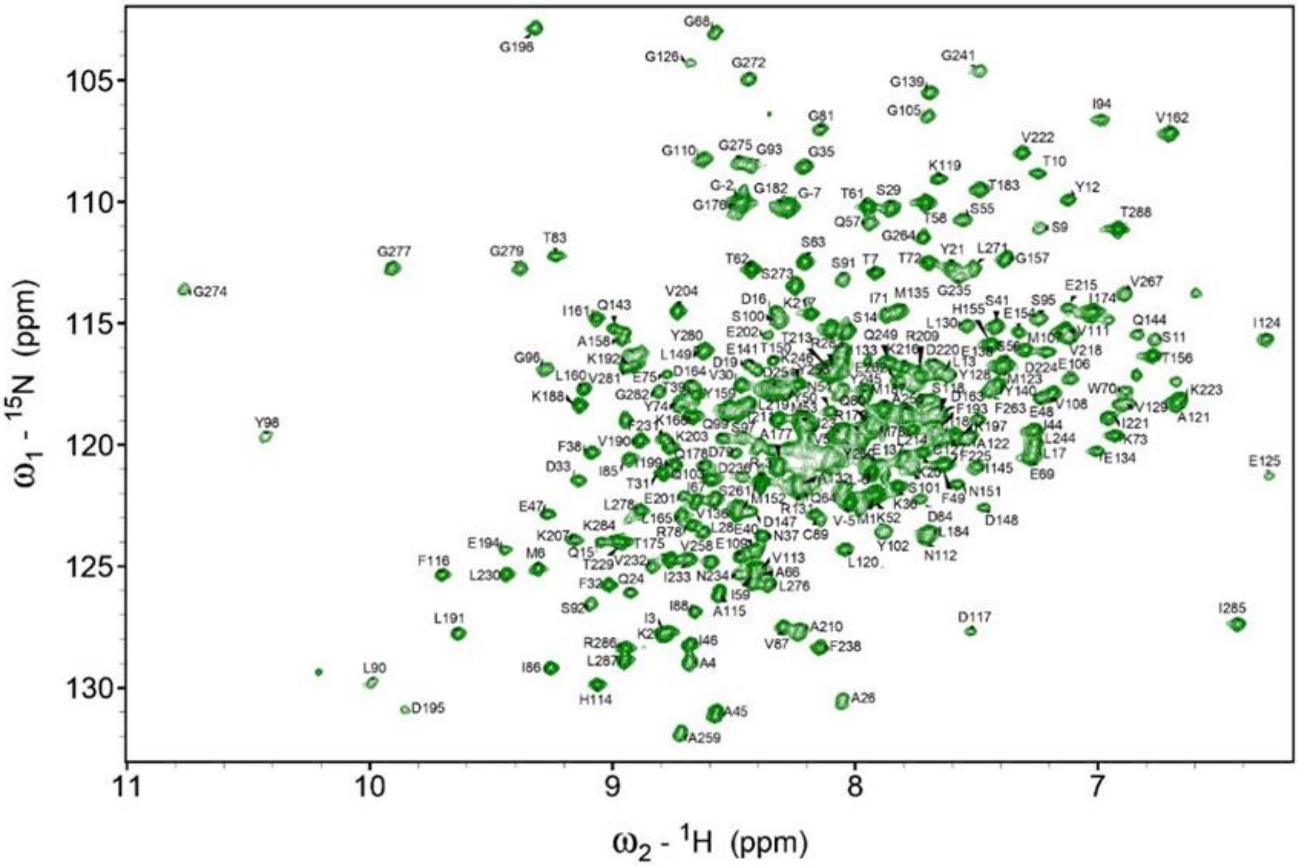

Figure S1. NMR spectroscopy of FakB1. Two-dimensional [^{15}N, ^{1}H] TROSY spectra of FakB1 at 293 °K with the residue assignments as indicated.

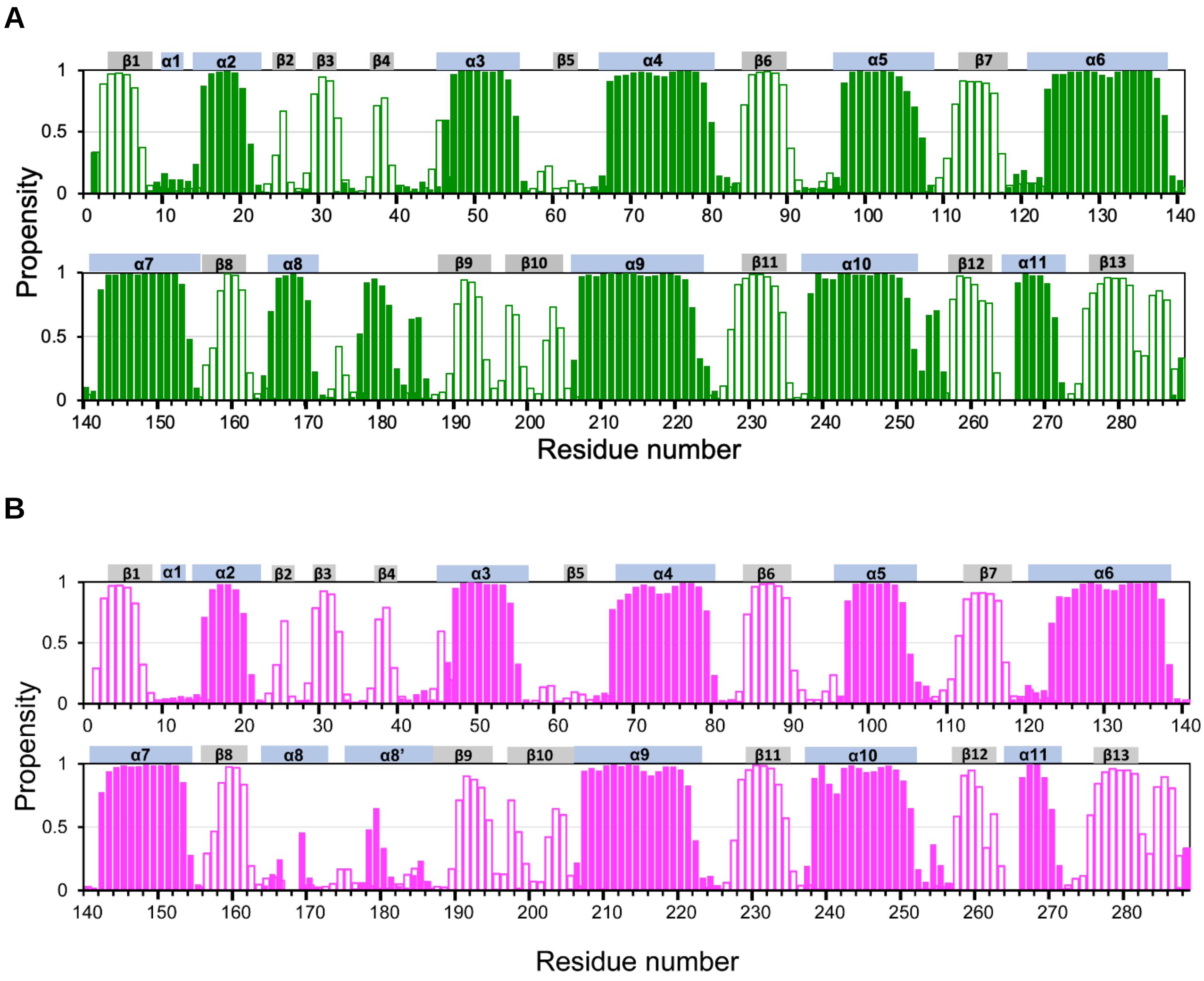

Figure S2. TALOS analysis of the NMR chemical shifts of FakB1 and FakB1(A121I). *A*, TALOS analysis of FakB1 at 293 °K. *B*, TALOS analysis of FakB1(A121I) at 293 K. The secondary structure elements based on the corresponding FakB1 and FakB1(A121I) crystal structures are shown above the TALOS analyses.

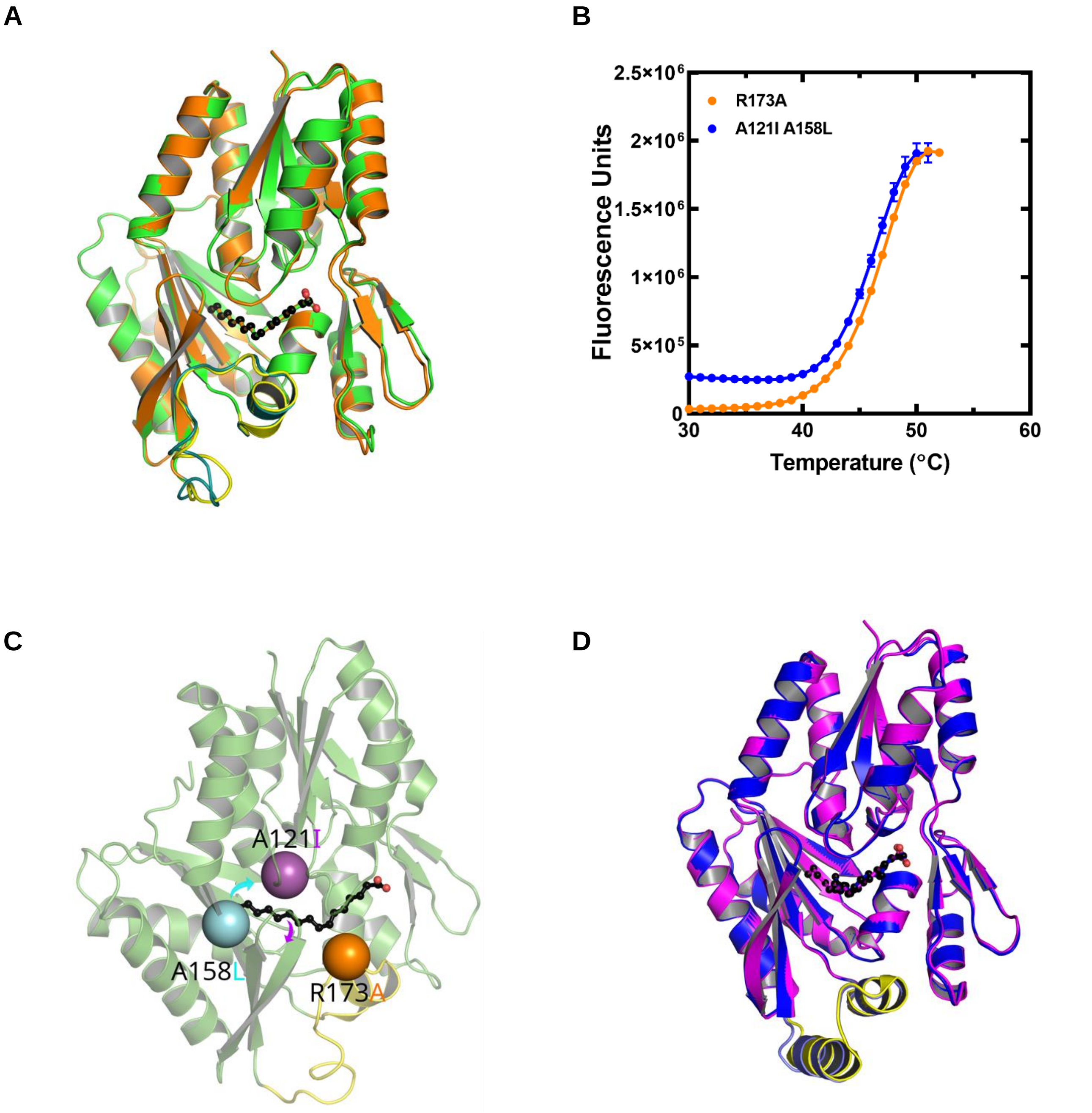

Figure S3. Key features of FakB1(A121I A158L) and FakB1(R173A). *A***,** overlay of the crystal structures of FakB1 (green), and FakB1(R173A) (orange) with palmitic acid. RMSD = 0.210 Å. Helix α8 and the α8-β9 loop region are highlighted in yellow (FakB1) and teal (FakB1(R173A)). *B*, thermal melting curves for FakB1(R173A) and FakB1(A121I, A158L) using SYPRO orange and 10 μM protein. The thermal transition for FakB1(R173A) was 45.9 ± 0.1 °C, and the transition for FakB1(A121I, A158L) was 45.4 ± 0.1 °C. *C*, locations of the A121I, A158L and R173A mutations in the FakB1 crystal structure. The arrows indicate how the introduction of bulky side chains press on the FA binding pocket. *D*, overlay of the crystal structures for FakB1(A121I) (magenta), and FakB1(A121I, A158L) (blue). Helices α8 and α8′ are highlighted in yellow (FakB1(A121I) and light blue FakB1(A121I, A158L). RMSD = 0.241 Å.

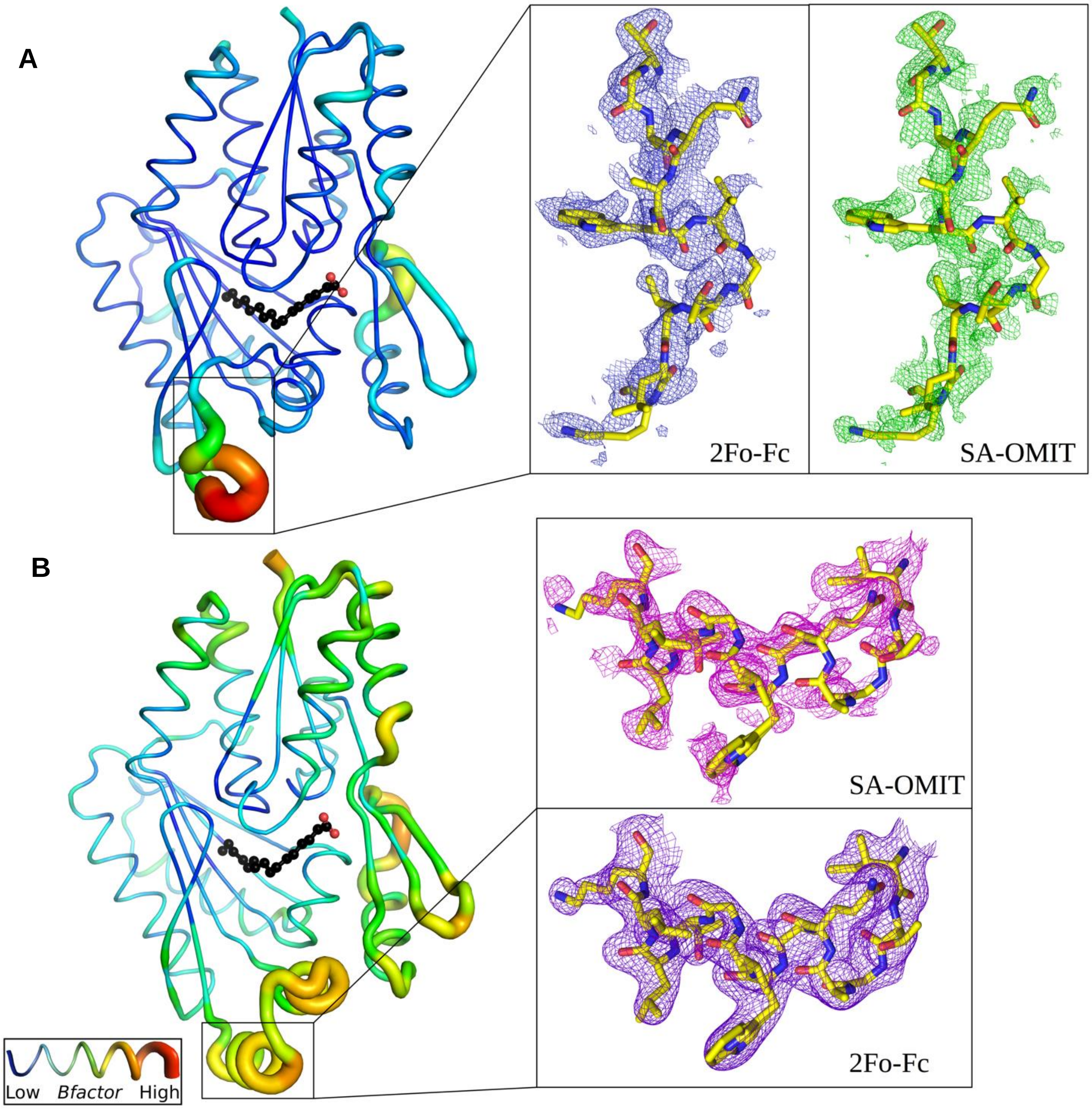

Figure S4. Quality of the FakB1 and FakB1(A121I) crystal structures. *A*, structure of FakB1 represented as a B-factor noodle/putty. The insets show the 2Fo-Fc and OMIT maps of the α8-β9 loop region. *B*, structure of FakB1(A121I) represented as a B-factor noodle/putty. The insets show the 2Fo-Fc and SA-OMIT maps for the helix α8′ region. 2Fo-Fc maps are contoured at 0.5 sigma level and SA-OMIT maps at 0.8 sigma level. The B-factor color scale is shown as noodle helix.

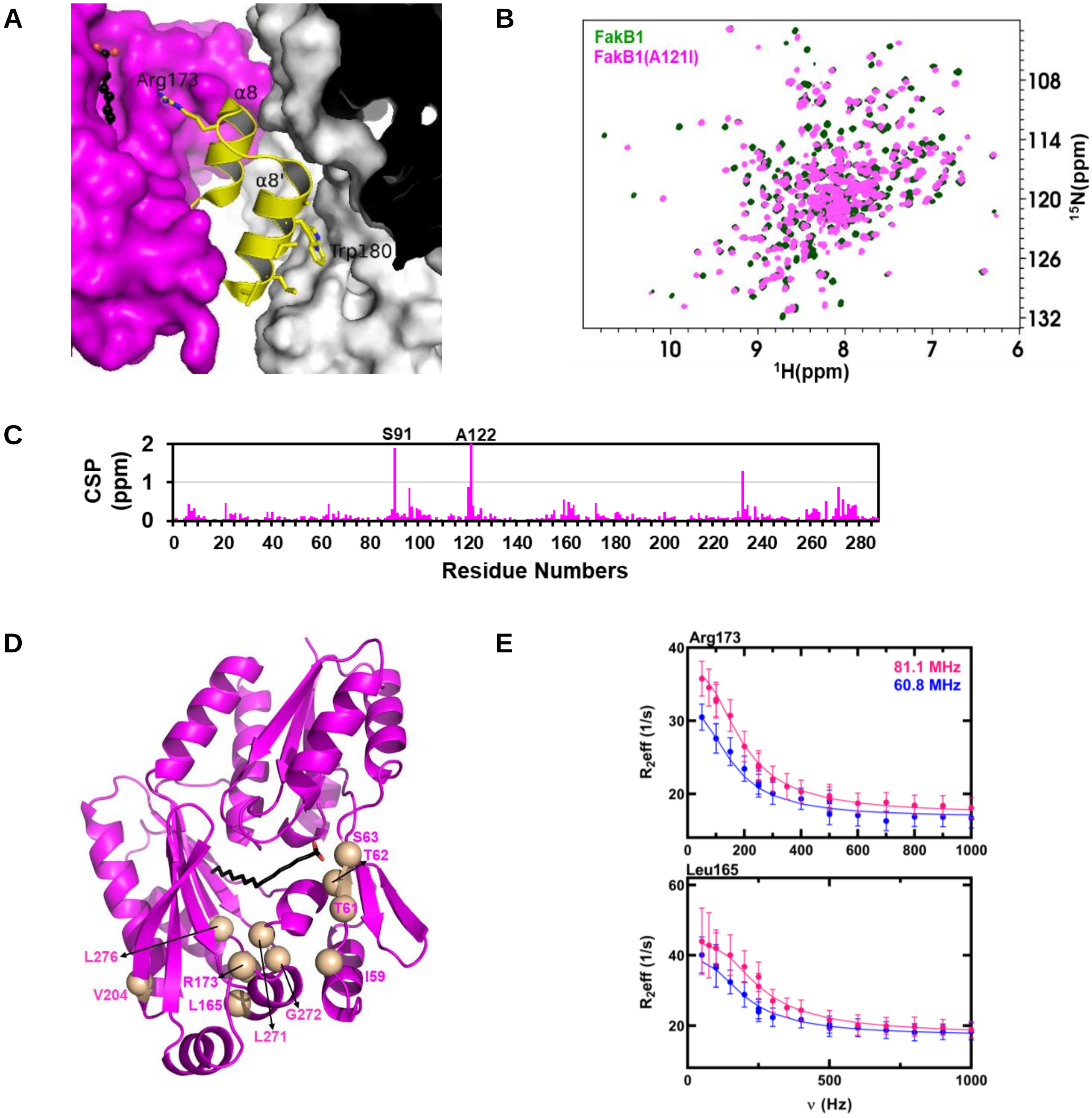

Figure S5. Characterization of FakB1(A121I). *A*, view of helices α8 and α8′ in FakB1(A121I) for molA within the crystal lattice illustrating the packing of helix α8′ against a hydrophobic patch on an adjacent monomer. *B*, overlay of the TROSY NMR spectra of FakB1 and FakB1(A121I). Asn42, Asn167, Leu168, Ser171, Thr206, His237, Asp240, and Ala268 could not be assigned in the FakB1(A121I) spectrum. *C*, chemical shift perturbations for the backbone amides of FakB1(A121I) with respect to FakB1. *D*, residues with measurable CPMG-RD exchange rates are indicated as yellow spheres mapped onto the FakB1(A121I) crystal structure. *E*, the two-state global fits (lines) are mapped onto the ^{15}N relaxation dispersion data at two field strengths for Arg173 and Leu165. Error estimates for R_2eff were obtained from duplicate measurements at 100, 250, and 500 Hz as described in Methods.

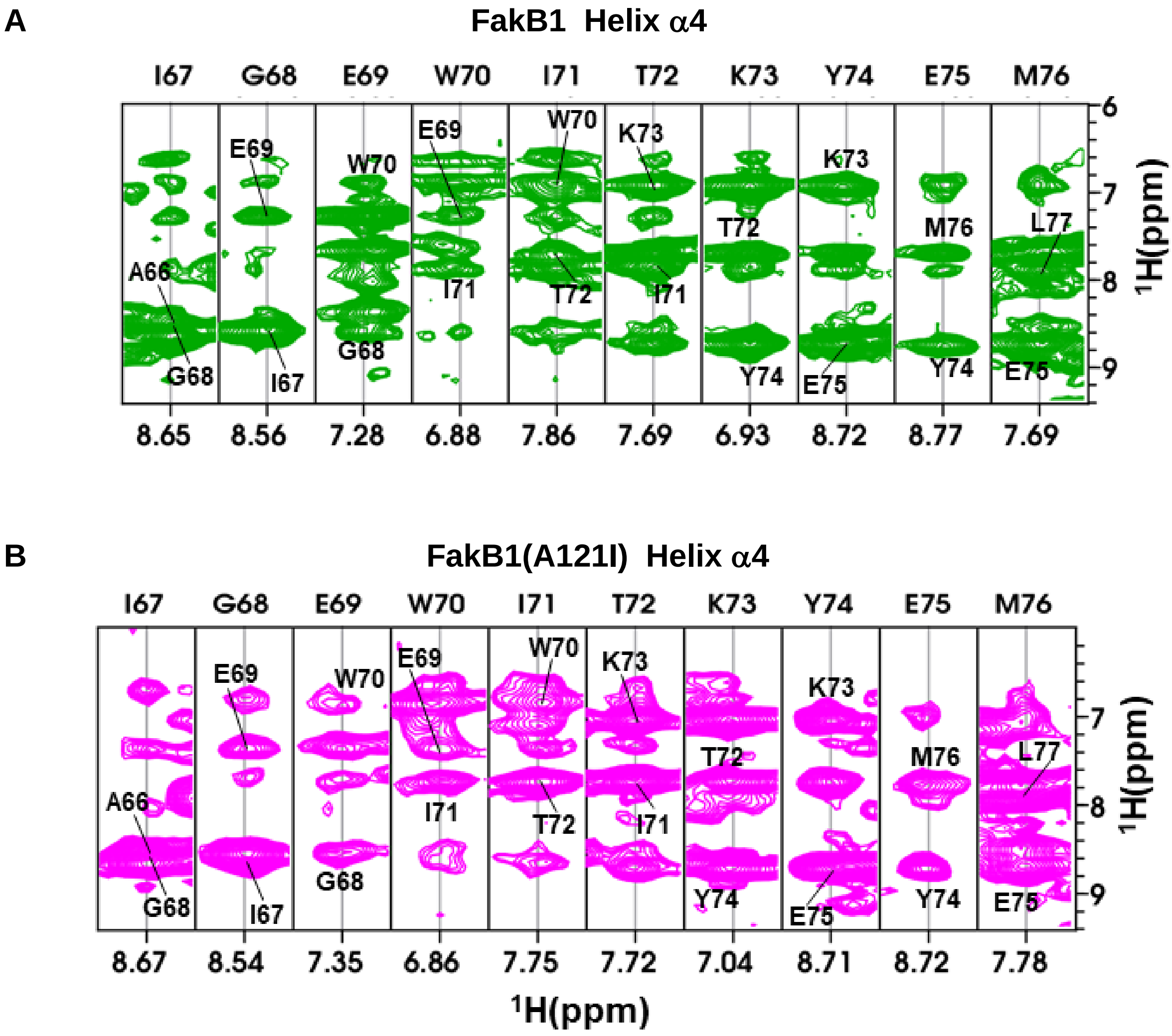

Figure S6. Structural analysis of helix α4 in FakB1 and FakB1(A121I) based on NOESY. *A*, NOE contacts between backbone amides within helix α4 in FakB1. *B*, NOE contacts between backbone amides within helix α4 in FakB1(A121I).

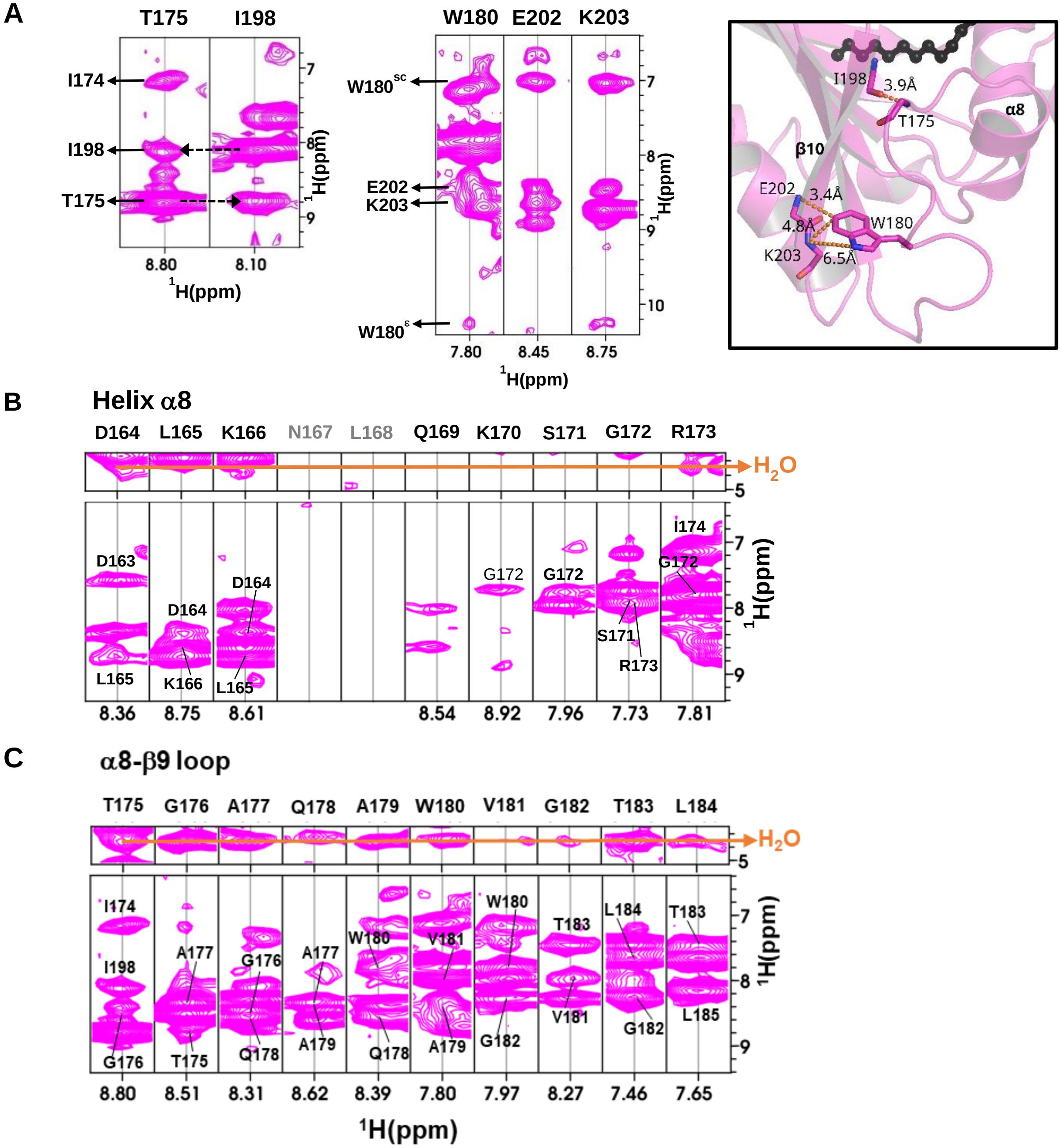

Figure S7. Structural analysis of FakB1(A121I) based on NOESY. *A*, NOE contacts between the α8-β9 loop of the dynamic region to residues on β10 in the main body of FakB1(A121I) are consistent with the closed conformation crystal structure. The NOE interactions (dotted orange lines) and computed distances from the NMR data are shown. *B*, sequential NOEs confirm the existence of helix α8 in solution and the exchange cross peaks with water are shown by the orange line. *C*, the water cross peaks in the region spanning Thr175-Leu184 are indicative of a structured loop, although the presence of sequential NOEs suggest the partial helical character of the loop centered on Ala179-Val181.

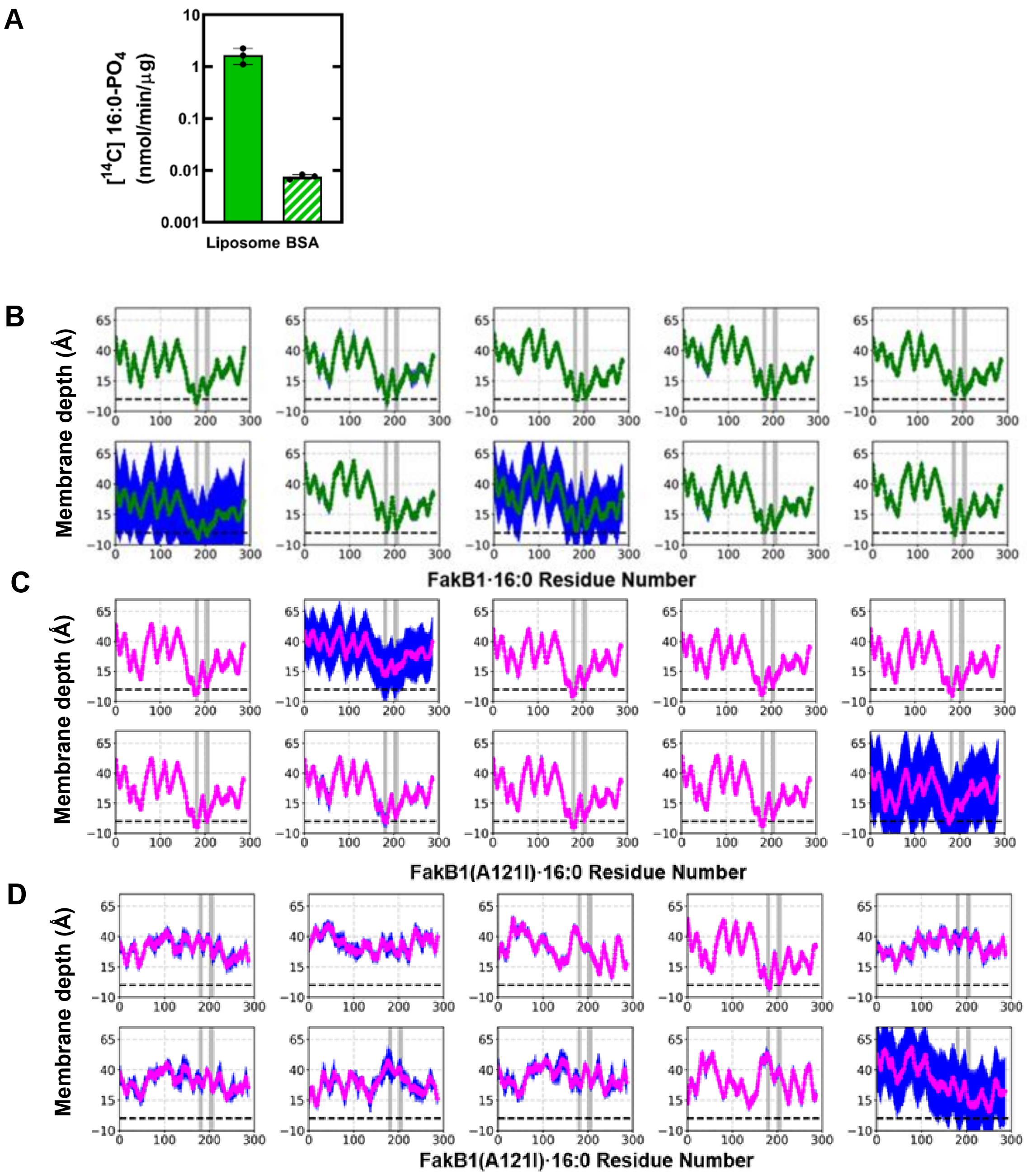

Figure S8. FakB1 exchange assay and the distances of individual residue FakB1 Cα carbons along the z-axis of the membrane calculated during the last 50 ns of HMMM MD simulations. *A*, FakB1 FA exchange assay using $[^{14}C]$16:0 presented as a BSA-16:0 complex or in PG liposomes. Mean values ± SD are shown; *n* = 3 independent experiments. *B*, ensemble-averaged Cα distances of FakB1 closed conformation association with PG bilayers. *C*, ensemble-averaged Cα locations of FakB1(A121I) open conformation binding to PG bilayers. *D*, ensemble averaged Cα distances for FakB1(A121I) to PC bilayers. *B-D*, we observed high error bars (standard deviations shown in blue) in some replicates due to membrane association taking place in last 50 ns of the simulation. Dashed lines are used to signify the level of the phosphate layer in the cis monolayer, which was used as a reference (z=0). Gray bars represent residues adjacent to or penetrating the bilayer.

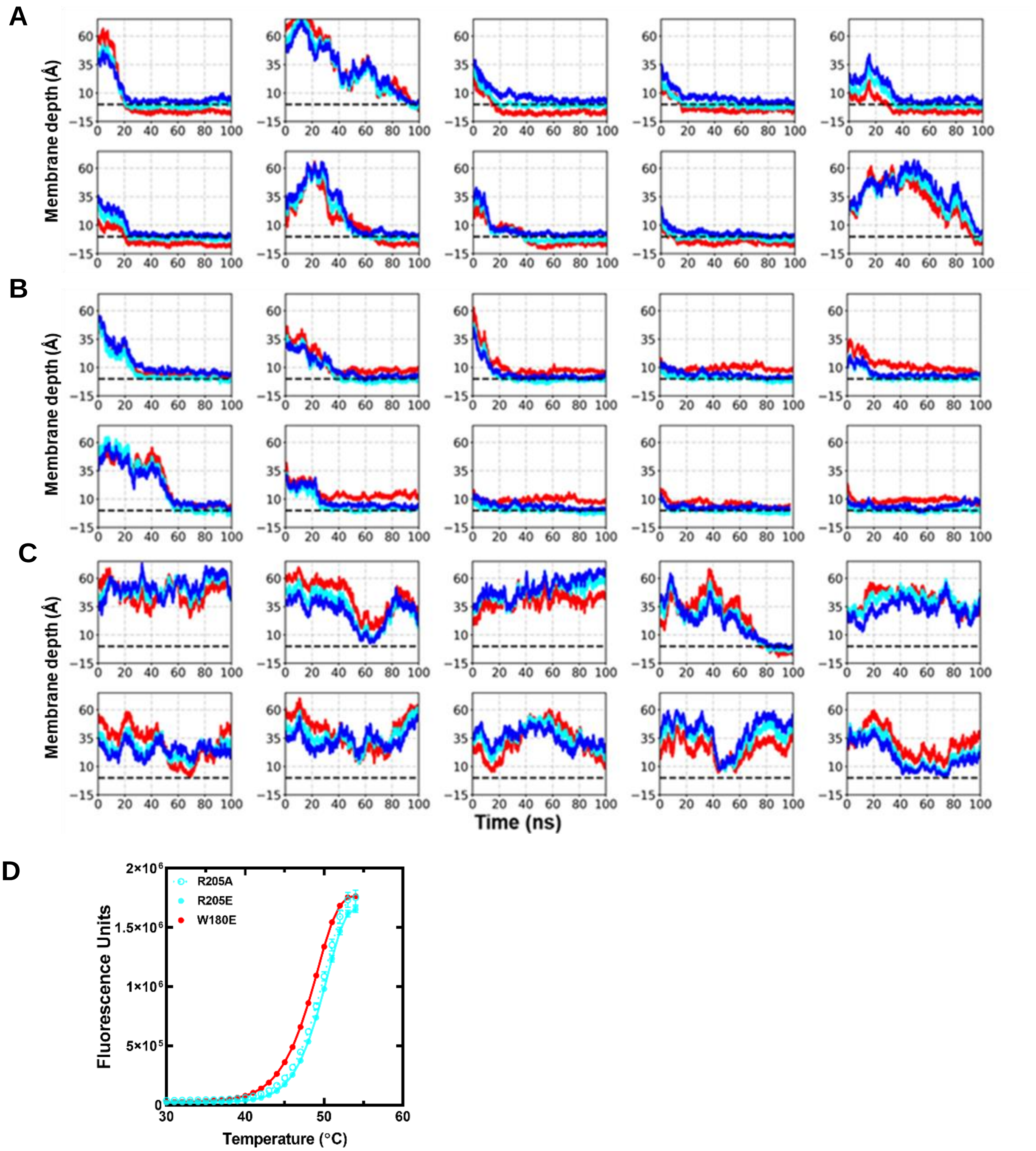

Figure S9. Time evolution of the center of mass (COM) of residue side chains for three sentinel FakB1 residues relative to the z-axis of the bilayer, stability of FakB1 mutants and overlay of FakB1 membrane insertion helices with those in mammalian FABP. Trp190 (red), Arg205 (cyan) and Arg209 (blue) along the membrane normal (z axis) are plotted as a function of time over 100 ns of 10 independent HMMM membrane binding simulations. *A***,** COM distances for membrane insertion of FakB1(A121I) open conformation in PG bilayers. *B*, COM distances for association of FakB1 closed conformation with PG bilayers. *C*, COM distances for FakB1(A121I) in PC bilayers. In A-C, dashed lines are used to signify the level of the phosphate layer in the cis monolayer, which was used as a reference (z=0). *D*, thermal melting curves using SYPRO orange and 10 μM protein. FkaB1(R205A) 49.2 ± 0.1 °C; FakB1(R205E), 49.2 ± 0.1 °C; and FakB1(W180E), 48.0 ± 0.01 °C.

Table S1. Sedimentation velocity c(s) analysis of *S. aureus* FakA and FakB1 proteins.

Sample	μM[a]	s_{20} (Svedberg)[b]	$s_{20,w}$ (Svedberg)[c]	Mw (Da)	f/f_0[d]
FakA	17.84	5.81 (78%)	6.10	131,730	1.49
FakB1	21.62	2.74 (86%)	2.88	32,999	1.24
FakB1(A121I)	16.14	2.75 (86%)	2.88	33,347	1.29
FakB1(A158L)	17.77	2.77 (95%)	2.91	32,740	1.24
FakA +	12.34	2.80 (11%)	2.93	48,858	1.72
FakB1	15.34	6.30 (72%)	6.61	182,488	1.72
FakA +	11.72	2.58 (9%)	2.71	43,546	1.61
FakB1(A121I)	13.42	6.22 (75%)	6.52	162,939	1.61
FakA +	12.48	2.62 (10%)	2.75	46,845	1.67
FakB1(A158L)	14.60	6.38 (76%)	6.70	177,974	1.67

[a]Total protein concentrations in μM.

[b]Sedimentation coefficient taken from the ordinate maximum of each peak in the best-fit c(s) distribution at 20 °C with percentage protein amount in parenthesis. Sedimentation coefficient (s-value) is a measure of the size and shape of a protein in a solution with a specific density and viscosity at a specific temperature. Values below 5% were not listed.

[c]Standard sedimentation coefficient (s20,w -value) in water at 20 °C.

[d]Molar mass values (MW) taken from the c(s) distribution that was transformed to the c(M) distribution. e[f] Best-fit weight-average frictional ratio values $(f/f_0)w$ taken from the c(s) distribution.

Table S2. Strains, plasmids, and primers used in this study.

Strains and Plasmids	*Description*	*Source*
**Strains**		
AH1263	USA300-0114, Erm-sensitive	Boles, 2010[a]
JLB31	*fakB1*:: ΦΝΣ Δ*fakB2* of strain AH1263	Parsons, 2014[b]
**Plasmids**		
pCS119	pCM28SarAP1promoter	Ericson, 2017[c]
pB1	pCS119 expressing *S. aureus* FakB1	Gullett, 2019[d]
pA121I	pCS119 expressing *S. aureus* FakB1(A121I)	This study
pA158L	pCS119 expressing *S. aureus* FakB1(A158L)	This study
pPJ597	pCS119 expressing *S. aureus* FakB1(W180E)	This study
pET15b	Expression vector	Novagen
pET28a	Expression vector	Novagen
pJLB11	*S. aureus* FakA in pET28a	Parsons, 2014[b]
pCS106	*S. aureus* FakB1 in pET15b	Parsons, 2014[b]
pPJ583	*S. aureus* FakB1(A121I) in pET15b	This study
pPJ584	*S. aureus* FakB1(A158L) in pET15b	This study
pPJ593	*S. aureus* FakB1(W180E) in pET15b	This study
pPJ594	*S. aureus* FakB1(R205A) in pET15b	This study
pPJ595	*S. aureus* FakB1(R205E) in pET15b	This study
pPJ631	*S. aureus* FakB1(R173A) in pET15b	This study
pPJ632	*S. aureus* FakB1(A121I A158L) in pET15b	This study
Primers		
FakB1(A121I) For	CTTCGATAGCAAACTGATAGCAATGATTGAAGGT TGC	This study
FakB1(A121I) Rev	GCAACCTTCAATCATTGCTATCAGTTTGCTATCG AAG	This study
FakB1(A158L) For	GCGTGAACATACCGGTCTCTATCTGATTGTTGAT G	This study
FakB1(A158L) Rev	CATCAACAATCAGATAGAGACCGGTATGTTCACG C	This study
CO A121I F	GTGTTAACGTTCATGCTTTTGATTCTAAACTTATT GCGATGATTGAAGGCT	This study
CO A121I R	AGCCTTCAATCATCGCAATAAGTTTAGAATCAAAA GCATGAACGTTAACAC	This study
CO A158L F	GATTTAACAAATATGCGTGAACATACAGGCTTAT ATTTGATTGTTGACGATTTAAAAAATCT	This study
CO A158L R	AGATTTTTTAAATCGTCAACAATCAAATATAAGCC TGTATGTTCACGCATATTTGTTAAATC	This study
B1 W180E – 1	GGGTGCCAACCTCTGCCTGTGCACCGGTAATAC	This study
B1 W180E – 2	GTATTACCGGTGCACAGGCAGAGGTTGGCACCC	This study
B1 R205E – 1	CTGAATTGCACGTTTTTTGGTTTCAACTTTTTCTT CCGGGATAATTTTGCCGTC	This study
B1 R205E – 2	GACGGCAAAATTATCCCGGAAGAAAAAGTTGAAA CCAAAAAACGTGCAATTCAG	This study
B1 R205A – 1	TGCACGTTTTTTGGTAGCAACTTTTTCTTCCGGG ATAATTTTGCC	This study
B1 R205A – 2	GGCAAAATTATCCCGGAAGAAAAAGTTGCTACCA AAAAACGTGCA	This study
CO B1 W180E Fwd	AATAACGTACCCACCTCAGCCTGTGCTCCTGTAA TTCGG	This study
CO B1 W180E Rev	CCGAATTACAGGAGCACAGGCTGAGGTGGGTAC GTTATT	This study

SaFakB1(R173A) F	ATCTGCAGAAAAGCGGTGCTATTACCGGTGCAC AGG	This study
SaFakB1(R173A) R	CCTGTGCACCGGTAATAGCACCGCTTTTCTGCAG AT	This study
B1(A121I A158L) F	GCGTGAACATACCGGTCTCTATCTGATTGTTGAT G	This study
B1(A121I A158L) R	CATCAACAATCAGATAGAGACCGGTATGTTCACG C	This study

[a]Boles, B. R., Thoendel, M., Roth, A. J., and Horswill, A. R. (2010) Identification of genes involved in polysaccharide-independent Staphylococcus aureus biofilm formation. *PLoS. ONE* **5**, e10146

[b]Parsons, J. B., Broussard, T. C., Bose, J. L., Rosch, J. W., Jackson, P., Subramanian, C., and Rock, C. O. (2014) Identification of a two-component fatty acid kinase responsible for host fatty acid incorporation by *Staphylococcus aureus*. *Proc. Natl. Acad. Sci. U. S. A.* **111**, 10532-10537

[c]Ericson, M. E., Subramanian, C., Frank, M. W., and Rock, C. O. (2017) Role of fatty acid kinase in cellular lipid homeostasis and SaeRS-dependent virulence factor expression in *Staphylococcus aureus*. *mBio* **8**, e00988-00917

[d]Cuypers, M. G., Subramanian, C., Gullett, J. M., Frank, M. W., White, S. W., and Rock, C. O. (2019) Acyl chain selectivity and physiological roles of *Staphylococcus aureus* fatty acid binding proteins. *J. Biol. Chem.* **294**, 38-49
